# Chemoenzymatic Synthesis of Fluorinated Cellodextrins Identifies a New Allomorph for Cellulose‐Like Materials**

**DOI:** 10.1002/chem.202003604

**Published:** 2020-12-09

**Authors:** Peterson de Andrade, Juan C. Muñoz‐García, Giulia Pergolizzi, Valeria Gabrielli, Sergey A. Nepogodiev, Dinu Iuga, László Fábián, Rinat Nigmatullin, Marcus A. Johns, Robert Harniman, Stephen J. Eichhorn, Jesús Angulo, Yaroslav Z. Khimyak, Robert A. Field

**Affiliations:** ^1^ Department of Biological Chemistry John Innes Centre Norwich NR4 7UH UK; ^2^ School of Pharmacy University of East Anglia Norwich Research Park Norwich NR4 7TJ UK; ^3^ Iceni Diagnostics Ltd. Norwich Research Park Innovation Centre Colney Lane Norwich Norfolk NR4 7GJ UK; ^4^ Department of Physics University of Warwick Coventry CV4 7AL UK; ^5^ Bristol Composites Institute CAME School of Engineering University of Bristol Bristol BS8 1TR UK; ^6^ School of Chemistry University of Bristol Bristol BS8 1TS UK; ^7^ Present address: Department of Chemistry and Manchester Institute of Biotechnology University of Manchester Manchester M1 7DN UK

**Keywords:** allomorphs, cellodextrins, chemoenzymatic synthesis, fluorine, soft-matter materials

## Abstract

Understanding the fine details of the self‐assembly of building blocks into complex hierarchical structures represents a major challenge *en route* to the design and preparation of soft‐matter materials with specific properties. Enzymatically synthesised cellodextrins are known to have limited water solubility beyond DP9, a point at which they self‐assemble into particles resembling the antiparallel cellulose II crystalline packing. We have prepared and characterised a series of site‐selectively fluorinated cellodextrins with different degrees of fluorination and substitution patterns by chemoenzymatic synthesis. Bearing in mind the potential disruption of the hydrogen‐bond network of cellulose II, we have prepared and characterised a multiply 6‐fluorinated cellodextrin. In addition, a series of single site‐selectively fluorinated cellodextrins was synthesised to assess the structural impact upon the addition of one fluorine atom per chain. The structural characterisation of these materials at different length scales, combining advanced NMR spectroscopy and microscopy methods, showed that a 6‐fluorinated donor substrate yielded multiply 6‐fluorinated cellodextrin chains that assembled into particles presenting morphological and crystallinity features, and intermolecular interactions, that are unprecedented for cellulose‐like materials.

## Graphical abstract

**Generating glycomaterials**: Enzymatic incorporation of singly and multiply fluorinated glucose residues into cellodextrin chains produces selectively fluorinated cellodextrins that self‐assemble into crystalline materials. Multiply 6‐fluorinated cellodextrin gave rise to an allomorph not previously reported for celluloses or cellulose‐like materials. Our findings highlight the potential of chemoenzymatic synthesis for generating novel glycomaterials of controlled molecular structure and morphology.

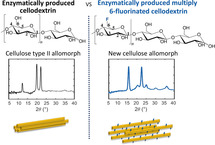

## Introduction

Cellulose is an abundant natural biopolymer used extensively in industry as a raw material for the production of paper, textile, food thickeners, dietary fibre, etc.[^1^, ^2^] The current use of cellulose increasingly involves nanosized cellulose particles (nanocellulose), which is a promising class of renewable material due to its intrinsic characteristics and potential for a broad range of industrial applications.[^3^, ^4^, ^5^] The development of nanocellulose‐based materials relies on assembly‐driven processes, the manipulation of which can have an impact on mechanical properties or bring additional functionality to the material.[^6^, ^7^, ^8^, ^9^, ^10^]

The production of cellulose nanocrystals and nanofibrillated cellulose, the main classes of nanocellulose, both rely on top‐down bioprocessing methodologies, based on the isolation of nanocellulose from cellulosic biomass, which requires high energy consumption.^[5]^ In addition, the functionalisation of nanocellulose to meet requirements for specific applications often requires harsh chemical conditions (i.e., strong acids and bases). As an alternative, enzymatic synthesis presents an attractive approach,[^11^, ^12^] enabling the bottom‐up preparation of site‐specifically modified oligo‐ and polysaccharides in a regio‐ and stereocontrolled manner.[^13^, ^14^, ^15^, ^16^] Specifically in relation to glucose‐based materials, glycoside phosphorylases (GPs)[^17^, ^18^, ^19^, ^20^, ^21^] have shown substantial potential for the synthesis of amylose‐ and cellulose‐like materials. In particular, cellodextrin phosphorylase (CDP, EC 2.4.1.49)[^22^, ^23^, ^24^] has emerged as a powerful tool for the synthesis of differently functionalised cellulose oligomers, giving rise to a variety of nanostructures (sheets,[^25^, ^26^] rods,^[27]^ or ribbons^[28]^) depending on the nature of the substrate.

The ability to systematically modify the structure of oligo‐ and polysaccharides presents new opportunities to gain insight into the hierarchical self‐assembly of carbohydrate‐based materials.^[29]^ In connection with this study, we had a need for the site‐specific introduction of probes into cellulose to report on local structure and solvation, and potentially to modulate material properties. Fluorine is well known for its unique physicochemical properties, such as small size, high electronegativity, great polarity and stability of the C−F bond.^[30]^ In addition, the absence of fluorine in biological systems and in the majority of materials makes the introduction of ^19^F nuclei a powerful reporter of local structure and environment. For instance, ^19^F NMR spectroscopy has been used to monitor crystallisation in nanoporous materials^[31]^ and fibril formation of intrinsically disordered proteins,^[32]^ to characterise polymeric biomaterials,^[33]^ and to map the interactions of fluorinated oligosaccharides with protein targets.^[34]^ Nonetheless, the use of fluorine remains under‐explored with respect to carbohydrate‐based materials.

The top‐down derivatisation of cellulose[^8^, ^35^] is complicated by solubility challenges, resulting in incomplete control of the sites and extent of fluorination.^[36]^ The bottom‐up chemical synthesis of structurally defined cellodextrins, including 3‐fluorinated compounds,^[37]^ has been achieved recently by automated chemical synthesis approaches.^[38]^ In this study, we wanted to investigate the impact of incorporating fluorine in place of the primary alcohols in cellulose, which are more accessible and have a fundamental role in the hydrogen‐bonding network that gives rise to the native cellodextrin structure (cellulose type II).^[39]^ Recognising the detrimental electronic impact of fluorination on sugar reactivity, we reasoned that enzymatic polymerisation per se may be inefficient: Kobayashi et al. note the reduced reactivity of 6‐fluorinated GlcNAc oxazoline towards chitinase‐mediated polymerisation, for instance.^[40]^ We therefore opted to exploit well‐studied cellodextrin phosphorylase (CDP) as it is known to produce DP 9 cellodextrin, which due to its limited water solubility results in antiparallel glucan chain association (cellulose II‐like material).^[22]^ Our expectation therefore was that the need for only a limited number of glycosylation events with CDP might still be achievable even allowing for the impact of fluorination on donor and/or acceptor substrate reactivity. Likewise, we expected to achieve a higher structural impact on the fluorinated cellodextrin produced from the modified donor, as it allows the introduction of multiple fluorine atoms along the chains. In addition, single site fluorination was also investigated to test the hypothesis of the effect of one fluorine atom per chain on the native cellodextrin structure. Furthermore, single site fluorination can be used as a potential probe for future multicomponent aggregation studies.

Herein, we enzymatically produced cellodextrins (EpCs) with different fluorination patterns. Monofluorinated EpCs (2F‐, 3F‐ and 6F‐EpC) were obtained by CDP‐mediated oligomerisation of α‐d‐glucose 1‐phosphate (Glc‐1P) as donor and deoxy‐fluoro‐cellobioses as acceptor substrates. Multiply 6‐fluorinated EpC (multi‐6F‐EpC) was prepared from 6‐deoxy‐6‐fluoro‐α‐d‐glucose 1‐phosphate (6F‐Glc‐1P) and cellobiose as donor and acceptor substrates, respectively. We demonstrate that the presence of a single fluorine atom per cellodextrin chain did not exert a substantial impact on the morphology and crystalline structure of the material, while the presence of multiple 6‐deoxy‐6‐fluoroglucose units yielded an unprecedented crystalline allomorph for a cellulose‐like material.

## Results and Discussion

### Enzymatic synthesis of fluorinated cellodextrins

#### Synthesis of 2‐, 3‐ and 6‐monofluorinated cellodextrins (2F‐EpC, 4; 3F‐EpC, 5; and 6F‐EpC, 6)

**Figure 1:**
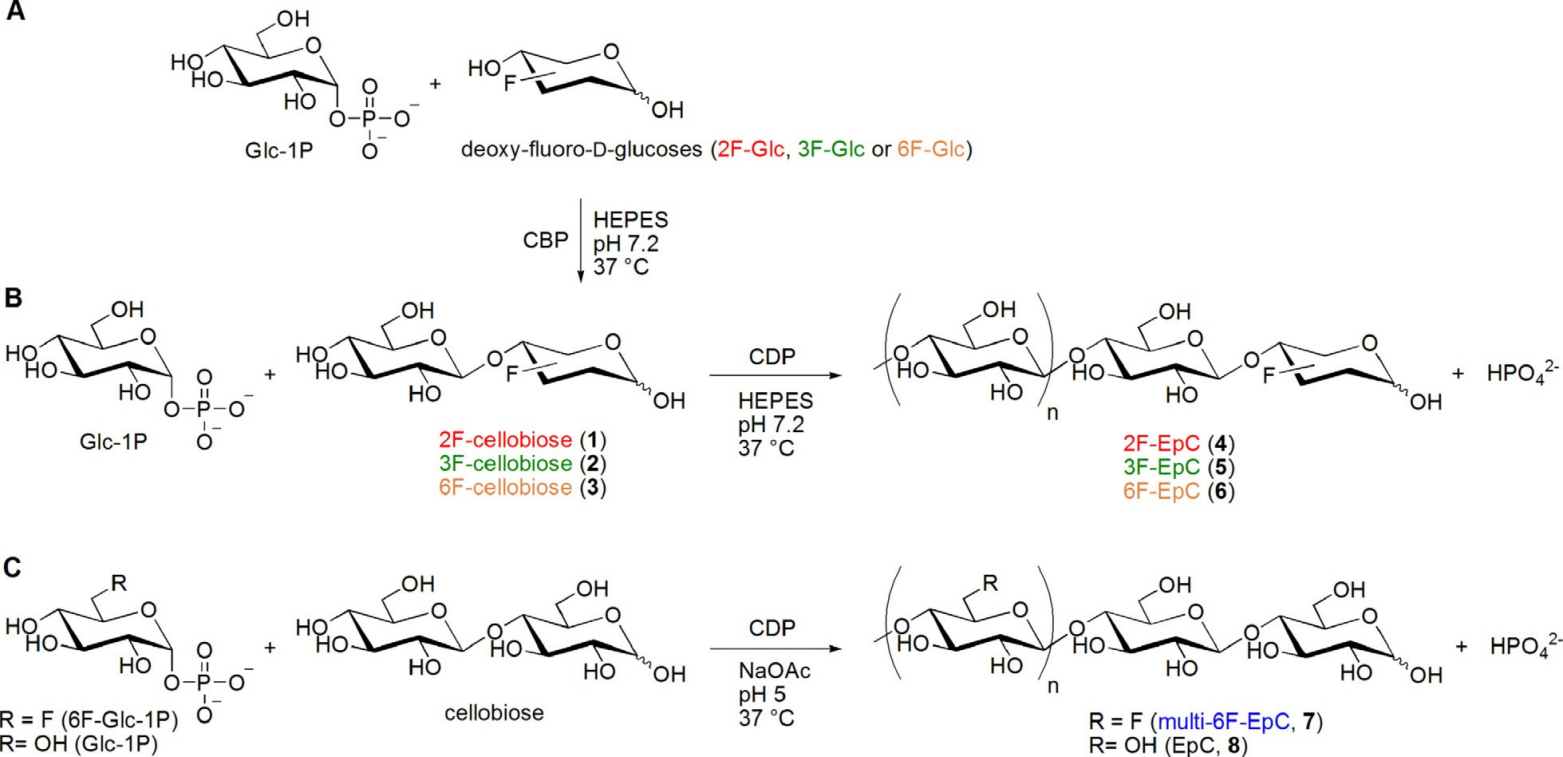
Enzymatic synthesis of fluorinated cellodextrins. A) Cellobiose phosphorylase (CBP)‐catalysed reaction of α‐d‐glucose 1‐phosphate (Glc‐1P) and deoxy‐fluoro‐d‐glucose (2F‐Glc, 3F‐Glc or 6F‐Glc), followed by B) cellodextrin phosphorylase (CDP)‐catalysed oligomerisation with Glc‐1P and monofluorinated cellobioses, to afford enzymatically produced fluorinated cellodextrins (2F‐EpC, **4**; 3F‐EpC, **5**; and 6F‐EpC, **6**). C) CDP‐catalysed reaction of 6‐deoxy‐6‐fluoro‐α‐d‐glucose 1‐phosphate (6F‐Glc‐1P) or Glc‐1P and cellobiose as acceptor to produce multiply 6‐fluorinated cellodextrin (multi‐6F‐EpC, **7**) or the parent enzymatically produced cellodextrin (EpC, **8**), respectively.

CDP uses glucose as an acceptor substrate only poorly, compared to cellobiose and longer cello‐oligosaccharides,[^22^, ^24^] to produce cellodextrins containing deoxy‐fluoro‐glucose at the reducing terminus (compounds **4**–**6**). We therefore initially used cellobiose phosphorylase (CBP; PRO‐GH94‐004) to synthesise monofluorinated cellobiose analogues (**1**–**3**; Figure 1 A) for use as acceptors for CDP (Figure 1 B). CBP was incubated at 37 °C with Glc‐1P (100 mm) and deoxy‐fluoro‐glucose (2F‐, 3F‐ or 6F‐Glc) (100 mm) for 16 h, at which point TLC showed approximately 80 % conversion into the disaccharides **1** and **3**, and ca. 60 % into **2**. The different conversion efficiencies may be rationalised based on a study of *Cellulomoinas uda* cellobiose phosphorylase,^[41]^ in which *k*_cat_/*K*_m_ values for 2F‐Glc (2.4 %), 3F‐Glc (0.013 %) and 6F‐Glc (31 %) acceptors are substantially lower than that of the parent Glc substrate, but all three compounds are indeed productive substrates. CBP was removed from the reaction mixture by affinity chromatography (His_6_ tag nickel column purification) and the desired products were purified by gel filtration chromatography. The purification successfully removed residual deoxy‐fluoro‐glucose acceptors, but small amounts of cellobiose required removal by HPLC to obtain compounds **1**–**3** (4–9 mg) in high purity for characterisation purpose (Figures S1–S3 in the Supporting Information). It is important to highlight that the monofluorinated cellodextrins **4**–**6** could be obtained in one‐pot reactions from the respective sugar‐1P and fluorinated glucoses without HPLC purification.

Once Glc‐1P consumption was almost complete in the CBP reactions, more Glc‐1P (4 equiv.) was added together with CDP and the reactions were incubated at 37 °C shaking for 16 h. A white precipitate was formed and isolated by centrifugation, followed by resuspension and washing with Milli‐Q water to remove enzyme, salts and any soluble sugars. A further 4 equiv. of Glc‐1P were added to the supernatant, and the CDP reaction was further incubated to produce more fluorinated EpCs. In this manner, monofluorinated cellodextrins were obtained (ca. 40 mg) with reasonable overall yield based on consumed fluoro‐glucose [47 % (2F‐EpC, **4**), 30 % (3F‐EpC, **5**), 32 % (6F‐EpC, **6**)]. MALDI‐TOF mass spectrometry analysis showed these materials to have an average DP of about 9, while the unsubstituted cellodextrin (EpC, **8**) produced under the same reaction conditions averaged ca. DP 8 (Figure S7). Traces of longer fluorinated cellodextrins were evident in the mass spectrometry data, which may reflect greater water solubility of the monofluorinated materials, thus resulting in further enzymatic extension. Solution‐state ^19^F NMR analysis in 1 m NaOD (Figure S4) showed two singlets for each material, reflecting reducing terminal anomers, with peaks at −195.21 and −195.26 ppm (2F‐EpC, **4**), −190.86 and −197.19 ppm (3F‐EpC, **5**) and −232.55 and −234.05 ppm (6F‐EpC, **6**).

#### Synthesis of multiply 6‐fluorinated cellodextrin (multi‐6F‐EpC, 7)

We also investigated CDP‐mediated oligomerisation using the chemically modified glucosyl donor 6F‐Glc‐1P (Figures S5 and S6)^[42]^ and cellobiose as acceptor (Figure 1 C) to achieve higher structural impact by placing multiple fluorine atoms along the cellodextrin (multiply 6‐fluorinated cellodextrin, multi‐6F‐EpC, **7**). The initial tests using 6F‐Glc as an acceptor to obtain a fully 6F‐substituted cellodextrin proved very slow and inefficient. Alternatively, CBP was tested with 6F‐Glc as an acceptor to generate a difluorinated cellobiose, which could be a better substrate for CDP. However, only trace amounts of the product were detected, prompting us to choose the natural acceptor cellobiose. CDP was incubated at 37 °C with 6F‐Glc‐1P (200 mm) and cellobiose (30 mm) for 72 h. The resulting white precipitate was isolated by centrifugation, followed by re‐suspension and washing with Milli‐Q water to give **7** with 64 % yield. ^19^F solution state NMR analysis of **7** dissolved in 1 m NaOD (Figure S4) showed one major singlet at −233.25 ppm, which may correspond to fluorine from the 6F‐Glc internal repeating units, and three smaller singlets at −233.29, −233.31 and −233.35 ppm from 6F‐Glc close to the reducing terminal and the non‐reducing terminal units. Analysis by MALDI‐TOF mass spectrometry revealed that multi‐6F‐EpC **7** (17 mg) had a higher average DP (ca. 10) than the parent EpC (ca. DP 8) and that longer chains, up to DP 15, could also be observed in the multiply 6‐fluorinated material (Figure S7). These data are comparable to the monofluorinated compounds and, more importantly, the presence of multiple fluorine atoms clearly had a higher impact on the DP of the cellodextrin products. The quantities of multiply 6‐fluorinated material obtained in these proof of concept studies enabled us to carry on to detailed structural characterisation at different length scales; scale up of enzymatic syntheses to provide materials for bulk physical properties assessment will be reported in due course.

### Morphological characterisation

#### Electron microscopy (EM) and atomic force microscopy (AFM)

**Figure 2:**
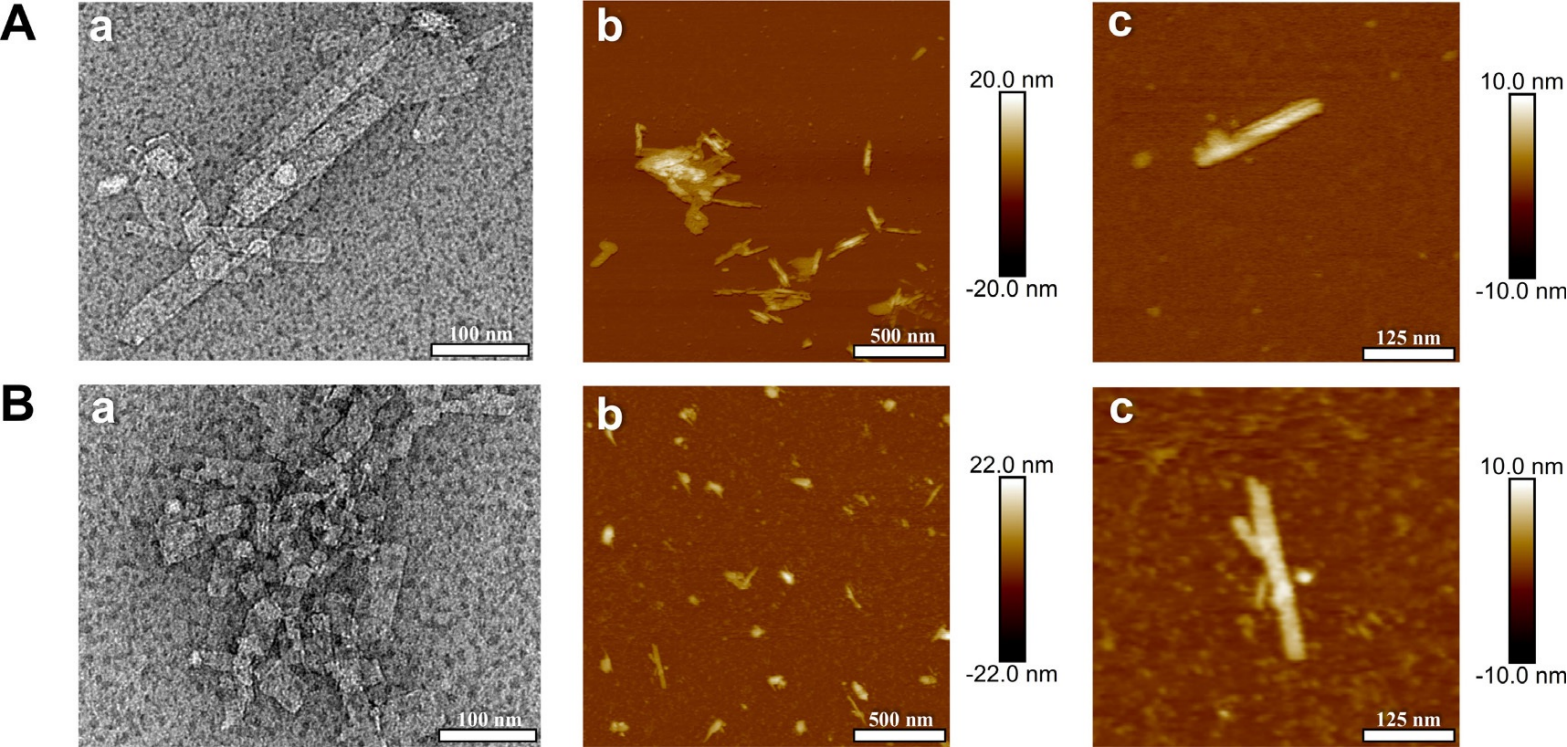
TEM (a) and AFM (b and c) images of A) EpC (**8**) and B) multi‐6F‐EpC (**7**). Scale bars are shown at the bottom of each image. The gradient bars next to b and c correspond to height measurements.

Transmission electron microscopy (TEM) was initially used to observe the morphological differences between EpC and fluorinated EpCs, which were prepared for analysis only by dilution of concentrated suspensions obtained after purification of precipitates formed during enzymatic synthesis. As expected, the TEM images of the monofluorinated 2F‐EpC (**4**), 3F‐EpC (**5**) and 6F‐EpC (**6**; Figure S8) show a very similar morphology to EpC (**8**, Figure 2 A, a). This crystalline sheet‐like morphology is well‐known for enzymatically synthesised cello‐oligosaccharides,[^22^, ^43^] including derivatised cellulose, such as acrylated cellulose^[26]^ and cellulose conjugated with oligo(ethylene glycol).^[28]^ On the other hand, multi‐6F‐EpC (**7**) particles formed predominantly into significantly shorter platelets (<100 nm length) (Figure 2 B, a). These differences were further confirmed by AFM imaging using samples prepared by depositing diluted sample suspensions on freshly cleaved mica (Figure 2 B, b and c). Although a few long platelets are present in multi‐6F‐EpC (**7**), their fraction is smaller than in EpC (**8**). As reported in the literature,[^22^, ^43^] the thickness of EpC (**8**) platelets was found to be ca. 5 nm. Similar thicknesses were observed for long platelets of multi‐6F‐EpC (**7**, Figure 2 B, c).

#### Long‐range structural characterisation by powder X‐ray diffraction (PXRD)

**Figure 3:**
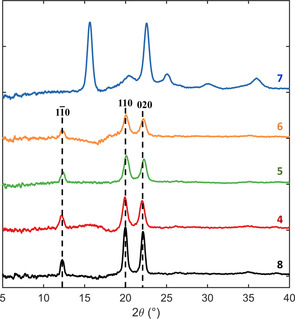
Powder wide‐angle X‐ray diffraction patterns of EpC (**8**, black), 2F‐EpC (**4**, red), 3F‐EpC (**5**, green), 6F‐EpC (**6**, orange) and multi‐6F‐EpC (**7**, blue).

The PXRD patterns of the monofluorinated 2F‐EpC (**4**), 3F‐EpC (**5**) and 6F‐EpC (**6**) are virtually indistinguishable from the diffraction pattern of EpC (**8**; Figure 3). This result indicates that the monofunctionalised cellodextrin‐like molecules arrange as a cellulose type II allomorph, with three intense and sharp peaks located at 2*θ*=12°, 20° and 23° (d‐spacings of 0.74, 0.44 and 0.39 nm, respectively) representing ($1\bar{1}0$ ), (110) and (020) planes.[^39^, ^44^] On the other hand, the experimental PXRD pattern reported for multi‐6F‐EpC (**7**) does not correspond to any allomorph previously described for cellulose[^44^, ^45^, ^46^, ^47^, ^48^, ^49^] (Figure 3). The pattern shows two well defined peaks at 2*θ*=15° and 23° (d‐spacings of 0.59 and 0.39 nm, respectively), as well as four different broad components at 2*θ*=21°, 25°, 30° and 36° (d‐spacings of 0.42, 0.36, 0.30 and 0.25 nm, respectively).

In order to verify possible similarities with previously reported cellulose structural organisations, we predicted and compared the PXRD spectra of multi‐6F‐EpC (**7**) to each known allomorph (Figure S9 and Table S1). Remarkably, the observed peak positions of multi‐6F‐EpC (**7**) are unique when compared to the diffraction patterns of the known allomorphs (Figures 3 and Figure S9, Table S1), hence demonstrating the formation of a new crystalline structure for this new cellulose‐like material.

### Molecular‐level characterisation

#### Raman spectroscopy

**Figure 4:**
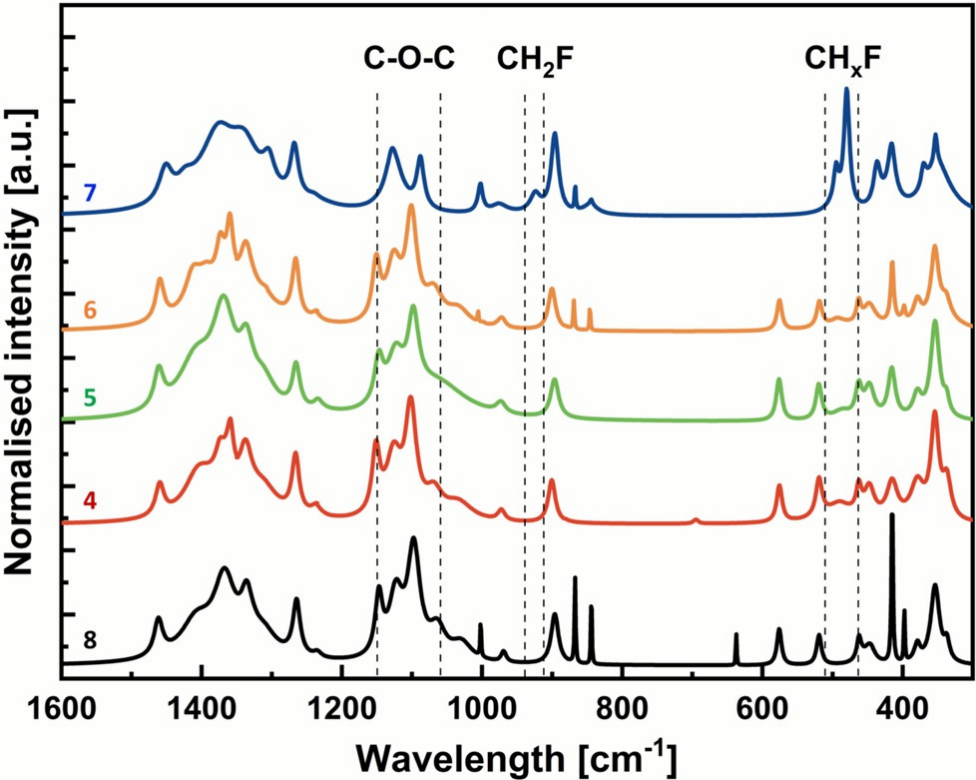
Deconvoluted and normalised Raman spectra for EpC (**8**, black), 2F‐EpC (**4**, red), 3F‐EpC (**5**, green), 6F‐EpC (**6**, orange) and multi‐6F‐EpC (**7**, blue). Dashed lines correspond to the boundaries of bands associated with C‐O‐C stretching (C‐O‐C) and the presence of fluorinated carbon groups (CH_2_F and CH_*x*_F). Each Raman spectrum represents the average of three Lorentzian‐deconvoluted spectra upon noise removal.

Figure 4 shows typical Raman spectra of 2F‐EpC (**4**), 3F‐EpC (**5**), 6F‐EpC (**6**), multi‐6F‐EpC (**7**) and EpC (**8**). The bands located at ca. 1462 (HOC and HCH stretching), 1265 (HCC and HCO stretching) and 576 cm^−1^ (heavy atom stretching) and the dominance of the band located at approximately 354 cm^−1^ over the band located at approximately 379 cm^−1^ (both heavy atom stretching) confirmed that EpC (**8**) arranges into a cellulose type II structure.^[50]^ Compounds **4**, **5** and **6** are very similar to **8**, as expected for a single fluorine atom (at the reducing end) per oligosaccharide chain. The weak band located at 487 cm^−1^ for the monofluorinated EpCs is probably an amalgamation of the 480 and 496 cm^−1^ bands as a result of the single fluorine present in each chain.

In contrast, the multi‐6F‐EpC (**7**) spectrum is significantly different owing to the presence of multiple fluorine atoms. Multi‐6‐fluorination results in new Raman bands located at 480, 496 and 924 cm^−1^. The first two bands correspond to the presence of CH_x_F, whilst the third relates to CH_2_F, confirming modification at the C6 position.^[51]^ It is likely that the presence of the additional bands is simply due to the more extensive presence of these groups within the multi‐6F‐EpC sample, since these are relatively more polar in nature, say compared to the C‐O‐C vibrations which being symmetric are present in all samples. Multi‐6‐fluorination also results in the shift of multiple bands, including 1462 to 1451 cm^−1^ and 1265 to 1268 cm^−1^, as well as the loss of others, such as the band located at 576 cm^−1^ (Table S2). Most significantly, the band associated with the glycosidic linkage located at 1097 cm^−1^ is shifted to 1088 cm^−1^. This provides some evidence that the crystal structure of the multi‐6F‐EpC material is neither cellulose type I nor type II. In any case, it should be noted that these changes most likely reflect differences in the packing of the oligosaccharide chains only, and do not involve conformational changes at the level of the glucose units. In this regard, quantum mechanics calculations carried out for fluorinated cellobiose showed that neither the puckering of the glucose units (^4^C_1_) nor the conformation of the glycosidic bonds are affected by the substitution of all hydroxy groups by fluorine atoms.^[52]^

#### Solid‐state nuclear magnetic resonance (SSNMR)

**Figure 5:**
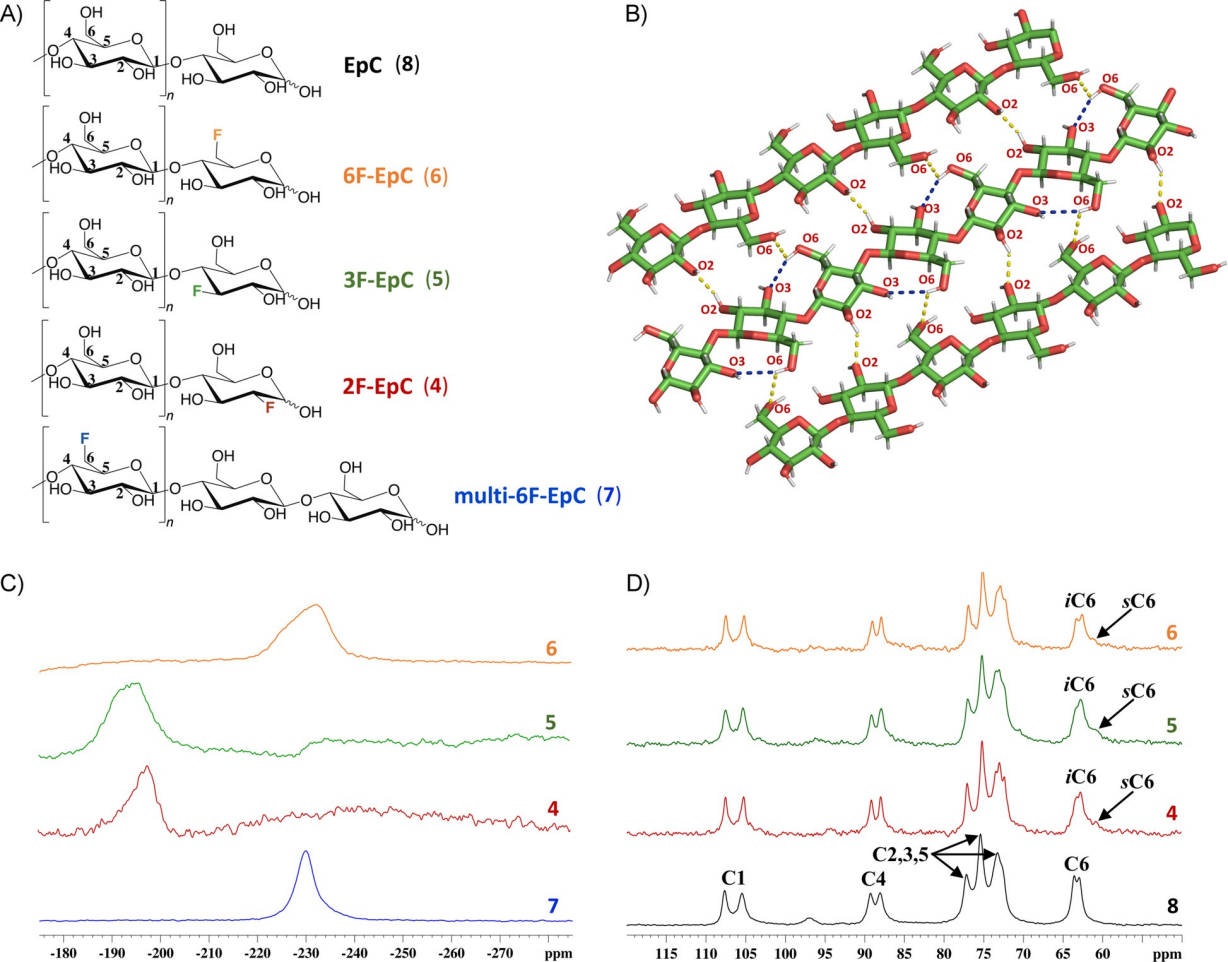
A) Chemical structures of unmodified EpC (**8**), monofluorinated 2F‐ (**4**), 3F‐ (**5**) and 6F‐EpC (**6**), and multi‐6F‐EpC (**7**). B) 3D model of the crystalline packing of the cellulose II allomorph based on the origin‐centre‐origin (o‐c‐o) chains.^[39]^ The O3–O6 intra‐chain (blue dashes) and O2–O2 and O6–O6 inter‐sheet (yellow dashes) hydrogen bonds are shown. It should be noted that the substitution of all ‐OH groups at C6 with fluorine atoms precludes the formation of O6–O6 inter‐sheet hydrogen bonds during self‐assembly. Note: the intra‐chain hydrogen bonds are only shown for the centre chain for simplicity.^[39]^ C) Direct detection ^19^F MAS NMR spectra of multi‐6F‐EpC (**7**, blue) and 2F‐ (**4**, red), 3F‐ (**5**, green) and 6F‐EpC (**6**, orange) powders, acquired at 60 kHz MAS rate and 800 MHz ^19^F frequency (20 T magnetic field). D) ^1^H–^13^C CP/MAS NMR spectra of EpC powder (**8**, black) acquired at 10 kHz MAS rate, and 2F‐ (**4**, red), 3F‐ (**5**, green) and 6F‐EpC (**6**, orange) 10 wt % dispersions acquired at 6 kHz MAS, and 100 MHz ^13^C frequency.

**Figure 6:**
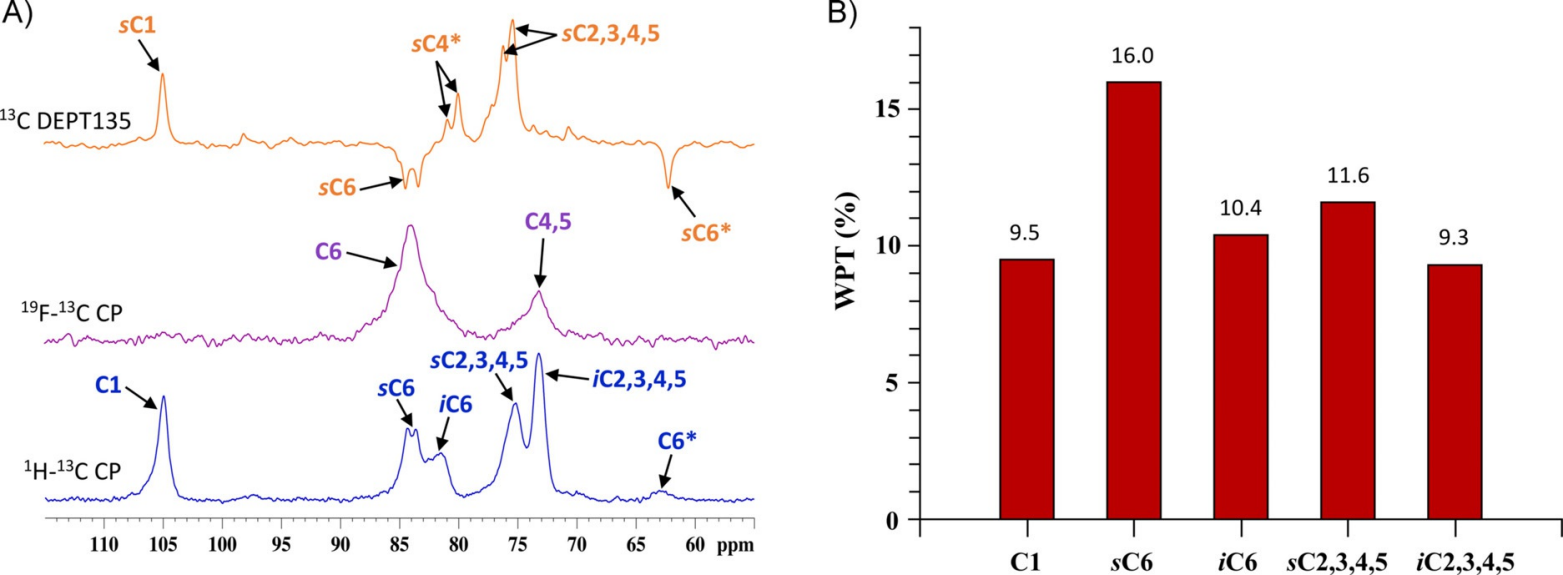
A) ^1^H–^13^C CP (blue) and ^1^H,^19^F‐decoupled ^19^F–^13^C CP (purple) NMR spectra of multi‐6F‐EpC (**7**) powder acquired at 60 and 15 kHz MAS spinning, respectively, and 212.5 MHz ^13^C frequency. The ^13^C DEPT135 spectrum of a 1 wt % dispersion of multi‐6F‐EpC (**7**) in D_2_O (orange) is shown for comparison. * Low‐intensity peaks corresponding to the non‐fluorinated glucose units of **7** at the reducing terminal of each cellodextrin chain. B) Bar graph showing the water polarisation transfer (WPT) factors [%] determined for each carbon peak of multi‐6F‐EpC 25 wt % hydrogel using a mixing time of 16 ms. The higher WPT factor observed for *s*C6 and *s*C2,3,4,5 compared to *i*C6 and *i*C2,3,4,5 demonstrates the increased solvation of the former, hence being assigned to surface domains. The individual WPT values appear on top of each bar.

Direct polarisation ^19^F NMR experiments (without ^1^H decoupling) were carried out at 60 kHz MAS rate for the 2F‐EpC (**4**), 3F‐EpC (**5**), 6F‐EpC (**6**) and multi‐6F‐EpC (**7**) (Figure 5 A). A single very broad and asymmetric peak was observed for the monofluorinated materials, centred at −190 (2F‐EpC, **4**), −197 (3F‐EpC, **5**) and −232 ppm (6F‐EpC, **6**), respectively, in good agreement with the solution ^19^F NMR data (Figure S4). 3F‐EpC (**5**) and 6F‐EpC (**6**) showed broad peaks, with line widths at half height of 11.9 and 9.4 kHz, respectively (Figure 5 C), while multi‐6F‐EpC (**7**) showed a sharper (3.8 kHz width at half height) Lorentzian‐shaped peak (Figure 5 C). ^1^H‐decoupled ^19^F (^19^F{^1^H}) NMR spectra of 3F‐EpC recorded at slower MAS rate showed an even broader ^19^F peak (Figure S10), indicating that i) fast MAS is more efficient at decoupling that radiofrequency decoupling (fast MAS decouples both ^19^F–^19^F homonuclear dipolar coupling as well as heteronuclear ^1^H–^19^F coupling), and ii) the large ^19^F line widths of 2F‐EpC (**4**), 3F‐EpC (**5**), 6F‐EpC (**6**) and multi‐6F‐EpC (**7**) in the fast MAS spectra (Figure 5 A) are mostly due to the large heterogeneity of ^19^F chemical environments. This can be easily understood considering that these materials assemble into particles with a specific crystalline packing (cellulose type II^44^ for 2F‐EpC (**4**), 3F‐EpC (**5**) and 6F‐EpC (**6**), and a new organisation for muti‐6F‐EpC (**7**); Figures 5 D and 6 A), and the ^19^F nucleus is extremely sensitive to chemical environment. Upon assembly of nanocellulose the ^19^F atoms of each cellulose chain can occupy any position within the nanofibril (surface, core, far from or nearby other fluorinated residues, etc.), hence presenting non‐equivalent environments within the packing of EpC (Figure 5 C). Assuming that ^19^F–^1^H dipolar interactions are reduced considerably at fast MAS, the peak broadening reflects a multitude of orientations sampled by the C−F bonds.

To characterise the structural organisation of 2F‐EpC (**4**), 3F‐EpC (**5**), 6F‐EpC (**6**) and multi‐6F‐EpC (**7**) materials at the molecular level, ^1^H–^13^C CP/MAS experiments were carried out. Each type of cellulose allomorph presents a characteristic ^13^C NMR fingerprint.[^53^, ^54^] Monofluorinated EpCs (**4**, **5** and **6**) 10 wt % dispersions showed the characteristic cellulose II ^1^H–^13^C CP fingerprint, typical of non‐modified EpC (**8**) (Figure 5 D). The only noticeable difference was the presence of a broad peak at about 61 ppm, which is characteristic of a surface/disordered population of C6 (*s*C6, Figure 5 D).[^54^, ^55^] Hence, the peak at 63 ppm represents the interior/ordered domains of C6 (*i*C6, Figure 5 D). The *s*C6 broad peak is typically observed in bacterial cellulose (BC), which consists of cellulose particles containing both I_α_ and I_β_ crystalline domains and disordered regions.^[55]^ Indeed, surface/disordered and interior/ordered domains are typically found in nanocrystalline cellulose,^[54]^ bacterial cellulose^[55]^ and plant cell walls.^[56]^ We note that the presence of a fluorine atom substituting the 3‐hydroxy group of glucose might affect the formation of the characteristic O3‐O6 intra‐chain hydrogen bond between adjacent glucose residues in cellulose II allomorph (Figure 5 B). On the other hand, the formation of the O2‐O2 and O6‐O6 inter‐sheet hydrogen bonds of cellulose II would be affected in 2F‐ and 6F‐EpC, respectively (Figure 5 B). Spectral deconvolution of the *s*C6 and *i*C6 peaks of ^1^H–^13^C CP/MAS NMR spectra enabled us to estimate the relative surface area (RSA) of the EpC particles constituting each material (Figure S11, Table S3). RSA values of about 16–23 % were obtained for the three monofluorinated EpCs, with 3F‐EpC showing the highest value (23 %; Table S3). Although the ^1^H‐^13^C CP/MAS NMR experiments were not fully quantitative, similar RSA values have been determined before for BC.^[55]^

The ^1^H–^13^C CP/MAS NMR spectrum of multi‐6F‐EpC (**7**) showed a pattern of peaks that does not correspond to either cellulose I, II or III allomorphs (Tables S4 and S5). This is evidenced by the appearance of C1 (105.9 ppm) as a singlet peak in **7**, which is a singlet in cellulose type I_α_ and III_I_, and a doublet in cellulose type I_β_, II and III_II_ (Table S4).[^47^, ^48^] The small shoulder observed at about 107 ppm cannot be assigned unambiguously to a specific structural feature. The different multiplicity of the peak corresponding to C1 indicates the presence of only one non‐equivalent anomeric carbon per unit cell in multi‐6F‐EpC (Figures 6 A and S12). This is different from unmodified EpC, which shows a doublet for C1 (Figure 5 D) due to the presence of two non‐equivalent anomeric carbons per unit cell. Also, the pattern of ^13^C chemical environments in multi‐6F‐EpC does not fully match any of the cellulose structures reported so far (Table S4). Hence, our solid‐state NMR data demonstrate that the inter‐chain interactions in multi‐6F‐EpC are different from non‐modified EpC or any other cellulose‐like structure.

Importantly, the PXRD pattern of multi‐6F‐EpC (**7**; Figure 3) does not correspond to any cellulose allomorphs reported so far (Table S1). Hence, **7** assembles into a crystalline organisation which is unprecedented for a cellulose‐type material. The formation of this novel structural motif is also supported by the new features observed in the Raman spectra, which do not correspond to either cellulose I or II (Figure 3, Table S4).

The combination of fast MAS ^1^H–^13^C CP, low MAS ^1^H,^19^F‐decoupled ^19^F–^13^C CP, water‐polarisation transfer (WPT) solid‐state NMR and ^13^C, COSY and HSQC solution NMR experiments enabled the assignment of the ^13^C spectrum of multi‐6F‐EpC (**7**; Figure 6) to be made. ^1^H,^19^F‐decoupled ^19^F–^13^C CP experiments enabled the assignment of C6, C4 and C5 peaks of the fluorinated residues (Figure 6 A). The highest intensity peak was assigned to C6 (83.8 ppm), as it is the carbon atom closest to 6F (1.3 Å). The peak at 73.1 ppm corresponds to C4 and C5 sites, based on their proximity to fluorine (Figures 6 A and S12), while C2 and C3 are too far away to cross‐polarise from fluorine effectively. ^13^C DEPT135, COSY and HSQC solution NMR experiments confirmed this assignment (Figure S13), with the methylene carbons of the fluorinated (C6) and non‐fluorinated (C6*) glucose units appearing in antiphase with respect to the CH carbons (Figure 6 A). Importantly, the ^13^C peaks at 81.9 and 73.1 ppm observed in the CP spectrum did not appear on the ^13^C DEPT135 or ^1^H–^13^C HSQC solution NMR experiments carried out for a diluted dispersion of multi‐6F‐EpC (**7**). Hence, these peaks most likely correspond to the immobile interior carbons (*i*C6 and *i*C2,3,4,5, respectively) that are too broad to be detectable by solution NMR. The solution‐NMR‐observed C6 and C2,3,4,5 peaks were therefore assigned to surface/disordered domains (*s*C6 and *s*C2,3,4,5, respectively). The assignment of *s*C6 and *i*C6 was further validated by water polarisation transfer CP (WPT‐CP) NMR experiments (Figure 6 B).^[57]^ The peak intensity in WPT‐CP experiments depends on the distance and relative mobility of bound water at the particle surface and the number of interacting water molecules at a particular site. Hence, peaks corresponding to surface domains will show faster WPT growth at short mixing times than interior domains, as we have recently observed for BC.^[55]^ At sufficiently long mixing times, WPT become homogeneous for both surface and interior domains due to the efficient spin diffusion. Figure 6 B shows the WPT factors for a 25 wt % dispersion of multi‐6F‐EpC (**7**) at 16 ms mixing time (under our experimental conditions, homogenisation of surface‐interior water polarisation transfer is achieved around 200 ms). A much higher WPT factor was observed for the *s*C6 (83.8 ppm) compared to the *i*C6 peak (81.8 ppm), confirming the assignment of *s*C6 and *i*C6 peaks to surface and interior domains, respectively (Figure 6 B). Also, *s*C2,3,4,5 showed higher WPT compared to *i*C2,3,4,5 (Figure 6 B), in agreement with solution NMR data where the *s*C2,3,4,5 and *i*C2,3,4,5 peaks are visible and invisible, respectively (Figure 6 A). Spectral deconvolution of *s*C6 and *i*C6 peaks of the ^1^H–^13^C CP spectrum acquired at 60 kHz, indicated that multi‐6F‐EpC (**7**) presents an RSA of ca. 54 % (Figure S11, Table S3). Although this experiment was not fully quantitative, the RSA determined for **7** is similar to what we have reported before for nanocrystalline cellulose.^[58]^

To summarise, we have demonstrated that the presence of multiple 6‐deoxy‐6‐fluoro glucose residues precludes the formation of the cellulose type II crystallinity and hydrogen bond patterns that defines EpC.^[39]^ To understand this at the molecular level, we should note that two different types of chains (centre, c, and origin, o) leading to three different types of hydrogen bond patterns (o‐o‐o, c‐c‐c and o‐c‐o) define the inter‐chain interactions of the EpC cellulose II structure. In particular, the O2‐H⋅⋅⋅O6, O6‐H⋅⋅⋅O2, O6‐H⋅⋅⋅O6 and O2‐H⋅⋅⋅O2 intermolecular hydrogen bonds characterise the cellulose II packing (Figures 5 B and S15). Thus, the presence of multiple 6‐deoxy‐6‐fluoro‐glucose residues per oligosaccharide chain will most likely prevent the formation of the O6‐H⋅⋅⋅O6 and O6‐H⋅⋅⋅O2 hydrogen bonds. In addition, the weaker character of the C‐F⋅⋅⋅H‐O hydrogen bonds and the partially hydrophobic nature of fluorine will probably affect the formation of the O2‐H⋅⋅⋅O6 hydrogen bonds, leading to the formation of different hydrogen bond interactions and/or partial structural disorder.

The unprecedented PXRD pattern of multi‐6F‐EpC clearly shows that this material presents a crystalline organisation different from any known cellulose‐like material (Figure 3). In addition, the Raman spectra confirm that multi‐6F‐EpC long‐range ordering does not correspond to either cellulose I or II (Figure 4). This, together with the solid‐state NMR data showing different inter‐chain interactions, indicates that multi‐6F‐EpC assembles into a novel crystalline packing defined by a distinct pattern of hydrogen bond interactions never reported before for a cellulose‐like structure. In addition, both the PXRD and NMR data show that the multi‐6F‐EpC particle network present both ordered and disordered domains.

## Conclusions

In this study, we have demonstrated the enzymatic incorporation of singly and multiply fluorinated glucose residues into cellodextrin chains. The OH‐to‐F substitution is tolerated by the cellodextrin phosphorylase, albeit with low efficiency. Nonetheless, we were able to produce selectively fluorinated cellodextrins, averaging about DP 9 in size, that self‐assemble into crystalline materials. Singly fluorinated cellodextrins display structural features reminiscent of cellulose II, as judged by solid‐state NMR, powder X‐ray diffraction and Raman spectroscopy. In contrast, multiply 6‐fluorinated cellodextrin gave rise to a new allomorph, not previously reported for either native celluloses or cellulose‐like materials. Advanced solid‐state NMR methods have enabled the detailed characterisation of these novel materials, deciphering the water‐exposed and interior chemical environments for different carbon sites. Our findings highlight the considerable potential of chemoenzymatic synthesis for generating novel glycomaterials of controlled molecular structure and morphology.

## Conflict of interest

The authors declare no conflict of interests.

## Supporting information

As a service to our authors and readers, this journal provides supporting information supplied by the authors. Such materials are peer reviewed and may be re‐organized for online delivery, but are not copy‐edited or typeset. Technical support issues arising from supporting information (other than missing files) should be addressed to the authors.

Supplementary
