# Supplementary material for: Chemoenzymatic Synthesis of Fluorinated Cellodextrins Identifies a New Allomorph for Cellulose‐Like Materials
Source: Chemistry. 2020 Dec 9;27(4):1374–82. doi: 10.1002/chem.202003604 (PMC7898601; doi:10.1002/chem.202003604)
Supplement: Supplementary file 1 — Supplementary [file CHEM-27-1374-s001.pdf]

Chemistry–A European Journal

Supporting Information

Chemoenzymatic Synthesis of Fluorinated Cellodextrins Identifies a New Allomorph for Cellulose-Like Materials**

Peterson de Andrade+,[a, g] Juan C. Muñoz-García+,[b] Giulia Pergolizzi+,[a, c] Valeria Gabrielli,[b] Sergey A. Nepogodiev,[a] Dinu Iuga,[d] László Fábián,[b] Rinat Nigmatullin,[e] Marcus A. Johns,[e] Robert Harniman,[f] Stephen J. Eichhorn,[e] Jesús Angulo,[b] Yaroslav Z. Khimyak,*[b] and Robert A. Field*[a, c, g]

Table of Contents

1. Materials and methods
1.1. General materials and methods
1.2. Expression and purification of cellodextrin phosphorylase (CDP)
1.3. Chemical synthesis of 6-deoxy-6-fluoro-α-D-glucose 1-phosphate (6F-Glc-1P)
1.4. Enzymatic synthesis of fluorinated cellodextrins
1.4.1 Synthesis of 2-, 3- and 6-monofluorinated cellobioses **1-3**
1.4.2 Synthesis of 2-, 3- and 6-monofluorinated cellodextrins (2F-EpC, **4**; 3F-EpC, **5** and 6F-EpC, **6**)
1.4.3 Synthesis of multiply 6-fluorinated cellodextrin (multi-6F-EpC, **7**)
1.5. Matrix-assisted laser desorption ionisation time-of-flight mass spectrometry (MALDI-TOF MS)
1.6. Electron microscopy (EM)
1.7. Atomic force microscopy (AFM)
1.8. Powder X-ray diffraction (PXRD)
1.9. Raman spectroscopy
1.10. Characterisation of cellodextrins by solution- and solid-state NMR

2. Results
2.1. Enzymatic synthesis of fluorinated cellobioses and cellodextrins
2.1.1. Nuclear magnetic resonance (NMR) characterisation
2.1.2. Matrix-assisted laser desorption ionisation time-of-flight mass spectrometry (MALDI-TOF MS) characterisation
2.2. Morphological characterisation
2.2.1. Transmission electron microscopy (TEM)
2.3. Long-range structural characterisation
2.3.1. Powder X-ray diffraction (PXRD)
2.4. Molecular characterisation: local structure
2.4.1. Raman spectroscopy
2.4.2. Solid-state and solution-state NMR spectroscopy

1. Materials and methods

1.1. General materials and methods

Chemicals were commercially obtained as reagent grade and used without any purification. Deoxy-fluoro-D-glucoses (2F-, 3F- and 6F-Glc) and α-D-glucose 1-phosphate disodium salt hydrate (Glc-1P) were purchased from Toronto Research Chemicals (Canada) and Sigma-Aldrich (UK), respectively. Cellobiose phosphorylase (CBP) (PRO-GH94-004) was kindly provided by Prozomix Limited (UK) and Milli-Q (MQ) H_2O was used to prepare all buffers. Thin-layer chromatography (TLC) was performed on pre-coated silica gel 60 F_{254} plates (Merck) and compounds were visualised by UV irradiation (λ 254 nm) and/or by spraying TLC with staining solution (2% orcinol w/v in $EtOH/H_2O/H_2SO_4$ 15:1:2 v/v/v) followed by heating. Biotage SP4 flash chromatography system was used for purification of protected monosaccharides using normal phase (pre-packed SNAP cartridges) and the monofluorinated cellobiose analogues were purified by HPLC (Thermo Scientific Dionex Ultimate 3000) on a Luna OH column (5 µm HILIC 200 Å, 250 × 10 mm, Phenomenex) using 5 mM ammonium formate buffer (5%) and acetonitrile (95%) at 5 mL/min in isocratic elution over 25 min. Detection was performed by charged aerosol detector (CAD) with power function 1.00, data collection rate 10 Hz and nebulizer temperature 25 °C. Products were lyophilised using a Labconco FreeZone Benchtop freeze dryer. ^{1}H, ^{13}C, ^{31}P and ^{19}F NMR spectra were recorded on a Bruker Avance III 400 MHz and/or Bruker Avance Neo 600 MHz spectrometers at 298 K. Chemical shifts recorded in D_2O are reported with respect to the solvent residual peak at 4.79 ppm in ^{1}H NMR. High resolution mass spectra were acquired in a Synapt G2-Si mass spectrometer (Waters, UK) using electrospray ionisation (positive or negative mode). Optical rotations were measured at 20 °C using a Perkin-Elmer Model 341 polarimeter.

1.2. Expression and purification of cellodextrin phosphorylase (CDP)

A recombinant plasmid (pET15b) containing the CDP gene from *Ruminiclostridium thermocellum* (YM4 strain) was transformed into *E. coli* BL21 (DE3) cells and grown as described previously.[1] Briefly, 1 L of LB medium containing the transformant and carbenicillin (100 µg/mL) was incubated at 37 °C with shaking (200 rpm) until OD_{600} around 0.6. Heterologous protein expression was induced by adding isopropyl β-D-1-thiogalactopyranoside (IPTG) to a final concentration of 1 mM and incubating for 4 hours at 30 °C with shaking (180 rpm). The cells were harvested by centrifugation (4,000 × g, 20 min), re-suspended in lysis buffer (50 mM HEPES, pH 7.5, 100 mM NaCl, EDTA-free protease inhibitor cocktail tablet, 0.02 mg/mL DNaseI), lysed by cell disruption (30 Kpsi, constant flow) and the supernatant containing the recombinant proteins was separated from cell debris by centrifugation (20,000 × g, 30 min). Proteins were purified at 4 °C using an ÄKTA pure FPLC system (GE Healthcare). The supernatant was loaded to a 5 mL HisTrap™ HP column (GE healthcare) pre-equilibrated with buffer A (50 mM Tris-HCl, pH 8, 50 mM glycine, 5% glycerol, 500 mM NaCl, 20 mM imidazole). The column was washed with buffer A to remove unbound proteins followed by elution of bound proteins with buffer B (50 mM Tris-HCl, pH 8, 50 mM glycine, 5% glycerol, 500 mM NaCl, 500 mM imidazole). Further purification was carried out by gel filtration chromatography (Superdex S200 16/600 column, GE Healthcare) with 20 mM HEPES, pH 7.5, 150 mM NaCl, 1 mL/min. Fractions containing CDP were pooled and concentrated using Amicon Ultra-15 Centrifugal Filter (30,000 MW cut off) and the enzyme concentration (5.7 mg/mL) was determined by NanoDrop™ spectrophotometer (Thermo Fisher Scientific, UK). The His-tag CDP was stored in aliquots at -80 °C until required.

1.3. Chemical synthesis of 6-deoxy-6-fluoro-α-D-glucose 1-phosphate (6F-Glc-1P)

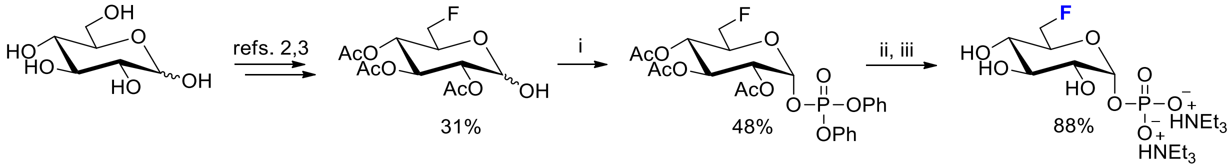

Reagent and conditions: i) *n*-BuLi, $(PhO)_2POCl$, THF, -78 °C to room temperature, overnight; ii) PtO_2, $H_{2(g)}$, EtOH, room temperature, 24 h; iii) Et_3N:H_2O:MeOH (1:3:7, v/v/v), room temperature, 48 h.

n-BuLi (2 mL of 1.6 M in hexane; 3.20 mmol; 1.2 eq.) was added dropwise to a solution of 6-deoxy-6-fluoro-2,3,4-tri-*O*-acetyl-D-glucopyranose (822 mg; 2.67 mmol; 1 eq.) (synthesised from D-glucose in five steps)[2,3] in anhydrous THF (40 mL) at -78 °C under N_2 atmosphere and stirred for 15 min. Diphenyl chlorophosphate (664 µL; 3.20 mmol; 1.2 eq.) was added dropwise and the reaction mixture was allowed to warm to room temperature overnight. The reaction was quenched with NH_4Cl saturated solution (30 mL) and partitioned with EtOAc (3 x 30 mL). The organic phase was washed with NaCl saturated solution (2 × 30 mL), dried over $MgSO_4$, filtered, concentrated under vacuum and purified by flash chromatography [cartridge SNAP 25g; solvent: Hexane/EtOAc; gradient: 0-20%, 20-20% and 20-30% (v/v); flow: 25 mL/min] to afford the product 6-deoxy-6-fluoro-2,3,4-tri-*O*-acetyl-α-D-glucopyranosyl 1-diphenylphosphate[4] in 48% yield (690 mg; 1.28 mmol). The deprotection steps of the synthesised phosphate (600 mg; 1.11 mmol) were performed with PtO_2 (50 mg; 0.22 mmol; 0.2 eq.) in absolute ethanol (25 mL) at room temperature under H_2 atmosphere for 48 h. After catalyst removal by filtration, the crude was concentrated under vacuum, dissolved in MeOH (20 mL) followed by addition of Et_3N (10 mL) and concentrated again. Lastly, the residue was dissolved in Et_3N:H_2O:MeOH (1:3:7, v/v/v) (45 mL) and stirred for 48 h at room temperature. After concentration under vacuum, the crude was dissolved in water and freeze dried to afford 6-deoxy-6-fluoro-α-D-glucose 1-phosphate triethylammonium salt[4] in 88% yield (457 mg; 0.98 mmol). ^{1}H NMR (400 MHz, D_2O) δ 5.49 (1 H, dd, $J_{1,P}$ 7.0 Hz, $J_{1,2}$ 3.5 Hz, H1), 4.83-4.58 (2 H, m, H6a, H6b), 3.97 (1 H, dd, $J_{5,F}$ 30.3 Hz, $J_{4,5}$ 10.6 Hz, H5), 3.78 (1 H, t, $J_{2,3} = J_{3,4}$ 9.5 Hz, H3), 3.60-3.51 (2 H, m, H4, H2), 3.20 (10 H, q, *J* 7.4 Hz, 2x $HN(C\underline{H}_2CH_3)_3$), 1.28 (16 H, t, *J* 7.3 Hz, 2x $HN(CH_2C\underline{H}_3)_3$). ^{13}C NMR (101 MHz, D_2O): δ 94.61 (C1), 82.80 (C6), 81.13 (C6), 72.56 (C3), 71.48 (C2), 71.36 (C5), 71.17 (C5), 68.27 (C4), 46.62 ($HN(\underline{C}H_2CH_3)_3$), 8.19 ($HN(CH_2\underline{C}H_3)_3$). ^{19}F NMR (^{1}H-decoupled, 376 MHz, D_2O): δ -236.30. ^{31}P NMR (162 MHz, D_2O): δ -1.07. HRMS (ESI): *m/z* calculated for $C_6H_{11}FO_8P$ $[M-H]^-$: 261.0181; found 261.0173.

1.4. Enzymatic synthesis of fluorinated cellodextrins

1.4.1. Synthesis of 2-, 3- and 6-monofluorinated cellobioses **1-3**

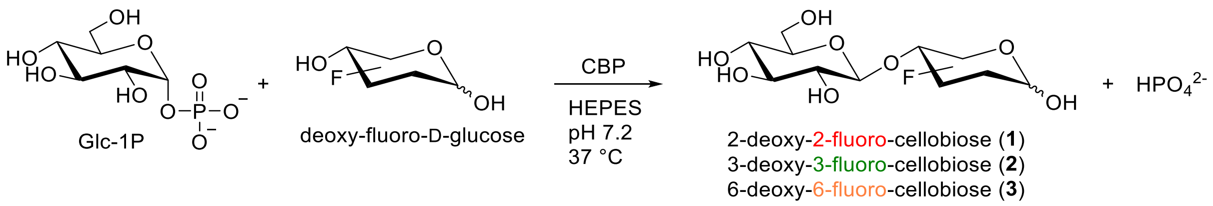

To a solution of deoxy-fluoro-glucose (F-Glc) (18.2 mg, 100 mM stock solution in Milli-Q (MQ) water, 1 mL, 1 eq., 13 mM final concentration) and Glc-1P (disodium salt hydrate, 30.4 mg, 100 mM stock solution in MQ water, 1 mL, 1 eq., 13 mM final concentration) in HEPES buffer (80 mM stock solution in MQ water, 5 mL, pH 7.2), was added cellobiose phosphorylase (CBP, 1 mg/mL stock solution, 1 mL, 1 mg) and the reaction was incubated at 37 °C shaking

for 16 h. The reaction mixture was centrifuged, and the supernatant was passed through HisTrap HP (1 mL) and High Q (5 mL) Bio-Rad columns to remove CBP and remaining Glc-1P, respectively. The pre-purified mixture was freeze dried, dissolved in 2 mL MQ water and purified by gel filtration chromatography (Toyopearl TSK HW40S, 1.6 × 90 cm, MQ water, 0.5 mL/min) followed by HPLC (Luna OH 5 µm HILIC 200 Å, 250 × 10 mm, Phenomenex, 5 mL/min in isocratic elution over 25 min, 5% 5 mM ammonium formate buffer and 95% acetonitrile) to afford the title compounds **1**-**3**:

2-Deoxy-2-fluoro-cellobiose (**1**).
Yield: 26% (9 mg; 0.029 mmol); Rf = 0.40 (isopropanol:NH_4OH:H_2O, 6:3:1); $[\alpha]_D^{20}$ +32.5 (c 1, CH_3OH). ^1H NMR (600 MHz, D_2O) δ 5.47 (0.45 H, d, $J_{1,2}$ 3.9 Hz, H1α), 4.95 (0.5 H, dd, $J_{1,2}$ 7.9 Hz, $J_{1,F}$ 2.4 Hz, H1β), 4.54 (1 H, two d, $J_{1',2'}$ 8.0 Hz, H1'), 4.48 (0.45 H, ddd, $J_{2,F}$ 49.5 Hz, $J_{2,3}$ 9.5 Hz, $J_{1,2}$ 3.9 Hz, H2α), 4.22-4.07 (1 H, m, H2β, H3α), 4.02-3.87 (3.5 H, m, H5α, H3β, $H6_a$β, $H6_a$α, $H6'_a$, $H6_b$α), 3.83 (0.5 H, dd, $J_{6a,6b}$ 12.4 Hz, $J_{5,6}$ 5.0 Hz, $H6_b$β), 3.78-3.72 (2 H, m, H4α, H4β, $H6'_b$), 3.67 (0.5 H, ddd, $J_{4,5}$ 9.9 Hz, $J_{5,6a}$ 5.0 Hz, $J_{5,6b}$ 2.2 Hz, H5β), 3.56-3.50 (2 H, m, H3', H5'), 3.47-3.42 (1 H, m, H4'), 3.37-3.32 (1 H, m, H2'). ^{13}C NMR (151 MHz, D_2O): δ 102.48 (C1'), 93.44 ($C1_β$), 93.21 ($C2_β$), 91.99 ($C2_β$), 90.55 ($C2_α$), 89.44 ($C1_α$), 89.32 ($C2_α$), 78.02 ($C4_α$), 77.97 ($C4_β$), 75.97 (C5'), 75.46 (C3'), 74.89 ($C5_β$), 73.11 (C2'), 72.76 ($C3_β$), 69.94 ($C5_α$), 69.92 ($C3_α$), 69.48 (C4'), 60.64 (C6'), 59.83 ($C6_β$), 59.68 ($C6_α$). ^{19}F NMR (^1H-decoupled, 376 MHz, D_2O) δ -199.08, -199.27. HRMS (ESI): *m/z* calculated for $C_{12}H_{21}FO_{10}Na^+$ $[M+Na]^+$: 367.1011; found: 367.1013.

3-Deoxy-3-fluoro-cellobiose (**2**).
Yield: 12% (4 mg; 0.012 mmol); Rf = 0.29 (isopropanol:NH_4OH:H_2O, 6:3:1); $[\alpha]_D^{20}$ +28.7 (c 1, CH_3OH). ^1H NMR (600 MHz, D_2O) δ 5.28 (0.5 H, t, $J_{1,2} = J_{1,F}$ 3.8 Hz, H1α), 4.77-4.66 (1 H, m, H3α, H1β), 4.64-4.51 (1.5 H, m, H3β, H1'), 4.04-3.96 (2 H, m, H5α, H4α, H4β, $H6_a$β), 3.94-3.90 (2 H, m, $H6_a$α, $H6'_a$, $H6_b$α), 3.88-3.82 (1 H, m, H2α, $H6_b$β), 3.72 (1 H, dd, $J_{6'a,6'b}$ 12.5 Hz, $J_{5,6'b}$ 5.9 Hz, $H6'_b$), 3.63-3.60 (0.5 H, m, H5β), 3.57 (0.5 H, ddd, $J_{2,F}$ 14.2, $J_{2,3}$ 9.1, $J_{1,2}$ 8.0 Hz, H2β), 3.53-3.49 (1 H, m, H3'), 3.48-3.44 (1 H, m, H5'), 3.42-3.37 (1 H, m, H4'), 3.33-3.28 (1 H, m, H2').^{13}C NMR (151 MHz, D_2O): δ 102.39 (C1'), 95.29 ($C3_β$), 95.05 ($C1_β$), 94.08 ($C3_β$), 93.73 ($C3_α$), 92.53 ($C3_α$), 91.99 ($C1_α$), 76.04 (C5'), 75.69 ($C5_α$), 75.56 (C3'), 75.49 ($C4_α$), 73.84 ($C5_β$), 73.27 (C2'), 72.76 ($C2_β$), 70.07 ($C2_α$), 69.86 ($C4_β$), 69.61 (C4'), 60.67 (C6'), 59.74 ($C6_β$), 59.61 ($C6_α$). ^{19}F NMR (^1H-decoupled, 376 MHz, D_2O) δ -192.35, -197.01 HRMS (ESI): *m/z* calculated for $C_{12}H_{21}FO_{10}Na^+$ $[M+Na]^+$: 367.1011; found: 367.1014.

6-Deoxy-6-fluoro-cellobiose (**3**).
Yield: 23% (8 mg; 0.023 mmol); Rf = 0.34 (isopropanol:NH_4OH:H_2O, 6:3:1); $[\alpha]_D^{20}$ +17.0 (c 1, CH_3OH). ^1H NMR (600 MHz, D_2O) δ 5.27 (0.55 H, d, $J_{1,2}$ 3.8 Hz, H1α), 4.96-4.85 (1 H, m, $H6_{a,b}$α), 4.76-4.65 (1.5 H, m, H1β, $H6_{a,b}$β), 4.52 (1 H, d, $J_{1',2'}$ 7.9 Hz, H1'), 4.15-4.06 (0.55 H, m, H5α), 3.94 (1 H, dd, $J_{6'a,6'b}$ 12.3 Hz, $J_{5,6'a}$ 2.2 Hz, $H6'_a$), 3.87 (0.55 H, t, $J_{2,3} = J_{3,4}$ 9.3 Hz, H3α), 3.82-3.72 (2.5 H, m, H5β, H4α, H4β, $H6'_b$), 3.69-3.65 (0.45 H, m, H3β), 3.62 (0.55 H, dd, $J_{2,3}$ 9.8 Hz, $J_{1,2}$ 3.8 Hz, H2α), 3.56-3.49 (2 H, m, H3', H5'), 3.47-3.43 (1 H, m, H4'), 3.37-3.31 (1.5 H, m, H2', H2β). ^{13}C NMR (151 MHz, D_2O): δ 102.62 (C1'), 95.90 ($C1_β$), 91.96 ($C1_α$), 82.39 ($C6_α$), 82.12 ($C6_β$), 81.28 ($C6_α$), 81.01 ($C6_β$), 77.80 ($C4_α$), 77.56 ($C4_β$), 75.97 (C5'), 75.48 (C3'), 74.10 ($C3_β$), 73.76 ($C2_β$), 73.33 ($C5_β$), 73.15 (C2'), 71.24 ($C3_α$), 71.13 ($C2_α$), 69.42 (C4'), 69.02 ($C5_α$), 60.56 (C6'). ^{19}F NMR (^1H-decoupled, 376 MHz, D_2O) δ -233.68, -234.24. HRMS (ESI): *m/z* calculated for $C_{12}H_{21}FO_{10}Na^+$ $[M+Na]^+$: 367.1011; found: 367.1014.

1.4.2. Synthesis of 2-, 3- and 6-monofluorinated cellodextrins (2F-EpC, 4; 3F-EpC, 5 and 6F-EpC, **6**)

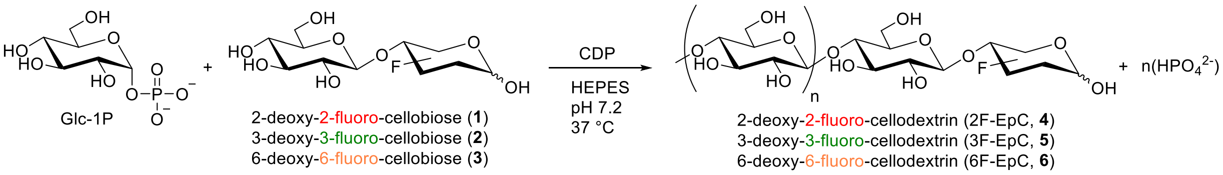

The monofluorinated cellodextrins were synthesised in a one-pot reaction. After CBP removal, more Glc-1P (110 mg, 4 eq.) was added together with cellodextrin phosphorylase (CDP, 0.9 mg/mL stock solution, 0.7 mL, 0.63 mg) and the reaction was incubated at 37 °C while shaking for 16 h. A white precipitate was formed and isolated by centrifugation, followed by re-suspension and washing with MQ water (3×). To the supernatant, more Glc-1P was added (90 mg) and the reaction incubated at 37 °C shaking for 12 h. A white precipitate was formed again and isolated by centrifugation, followed by re-suspension and washing with MQ water (3×). The precipitates were combined to give reasonable final yields [47% 2F-EpC (**4**), 30% 3F-EpC (**5**) and 32% 6F-EpC (**6**)]. The white solid was analysed by MALDI-ToF (DP8 on average) and solution-state ^{19}F NMR (^{1}H-decoupled, 376 MHz, 1 M NaOD) δ -195.21 and -195.26 ppm (**4**); -190.86 and -197.19 ppm (**5**); -232.55 and -234.05 ppm (**6**).

1.4.3. Synthesis of multiply 6-fluorinated cellodextrin (multi-6F-EpC, 7)

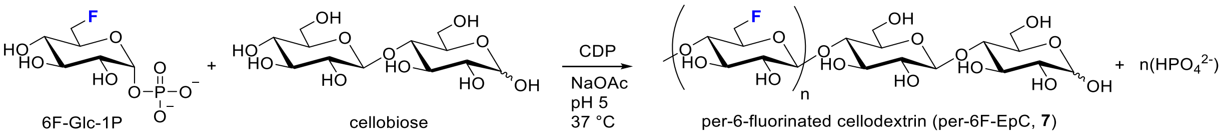

Cellodextrin phosphorylase (CDP, 5.7 mg/mL stock solution, 300 µL, 1.7 mg) was added to a solution of cellobiose (5.5 mg, 16.1 µmol, 1 eq.) and 6-deoxy-6-fluoro-α-D-glucose 1-phosphate (6F-Glc-1P) (triethylammonium salt, 45 mg, 96.6 µmol, 6 eq.) in 500 µL NaOAc buffer (200 mM, pH 5.0) and the reaction was incubated at 37 °C for 24 h with shaking (300 rpm). A white precipitate started forming and more CDP was added (1-fold) and the reaction continued in the same conditions for 48 h (72 h in total). The white precipitate was isolated by centrifugation, re-suspended and washed with MQ water (4×) and freeze dried to afford the multiple 6-fluorinated cellodextrin **7** in 64% yield (17 mg; 10.3 µmol). The white solid was analysed by MALDI-ToF (DP10 on average) and solution-state ^{19}F NMR (^{1}H-decoupled, 565 MHz, 1 M NaOD) δ -233.25, -233.29, -233.31, -233.35 ppm.

1.5. Matrix-assisted laser desorption ionisation time-of-flight mass spectrometry (MALDI-TOF MS)

Samples suspended in MQ water were mixed with equal volume of 2,5-dihydroxybenzoic acid (DHB) matrix (10 mg/mL in 30% acetonitrile in MQ water), spotted on a target plate (Bruker MTP 384 Polished Steel TF Target) and analysed on an Autoflex™ Speed MALDI-TOF/TOF mass spectrometer (Bruker Daltonics™ GmbH, Coventry, UK). The instrument was controlled by a flexControl™ (version 3.4, Bruker) method optimised for peptide detection and calibrated using peptide standards (Bruker). All spectra were processed with flexAnalysis™ (3.4, Bruker).

1.6. Electron microscopy (EM)

Transmission Electron Microscopy (TEM) images were viewed on a Thermo Fisher Talos F200C transmission electron microscope at 200kV (Thermo Fisher UK Ltd, Cambridge, UK) using a Gatan OneView 4k X4K digital camera (Gatan, Abingdon, UK) to record DM4 files. 400 mesh EM Resolution Formvar/Carbon coated copper grids were glow discharged, the samples were suspended in 200 µL MQ water and 5 µL drop was pipetted onto the grid for 1 minute. The excess was then blotted off with filter paper and 5 µL of 2% uranyl acetate was pipetted onto the grid for 30 seconds then blotted off with filter paper and allowed to dry.

1.7. Atomic force microscopy (AFM)

Samples of fluorinated cellodextrins were deposited onto freshly cleaved mica substrates by drop-casting of diluted suspensions (0.02 mg/ml). Cast drops were allowed to dry before SPM investigation in an ambient environment. SPM measurements were conducted using a Multi-Mode VIII microscope with Nanoscope V controller operating under non-resonant PeakForce feedback control (Bruker, CA, USA). SCANASYST-FLUID+ cantilevers were employed with nominal spring constants of 0.7 N/m (Bruker, CA, USA). Real-time analysis of the force-separation curves were collected as part of the Peakforce control mechanism allowing the physical tip-sample interaction forces and sample topography to be mapped concurrently.

1.8. Powder X-ray diffraction (PXRD)

X-ray diffraction data were collected using a single crystal diffractometer (Rigaku Synergy S, Cu X-ray tube, 50kV-1mA) with Cu Kα radiation (λ = 0.154 nm). Samples of enzymatically produced cellodextrin (EpC), 2F-EpC, 3F-EpC, 6F-EpC and multi-6F-EpC were placed in a 96-well plate and analysed using an XtalCheck screening plate mounted on the diffractometer, and each plate was covered with tape. The collected diffraction images were integrated between diffraction angles (2Θ) 5 and 40°. To account for the scattering contribution of the plate and tape, an empty plate covered with tape was used as reference. Hence, the corrected PXRD intensities (I_{cor}) of each sample were obtained by subtraction of the reference intensity (I_{ref}) to the observed intensity (I_{obs}), as expressed by Eq. S1:

$$I_{cor} = I_{obs} - I_{ref} \qquad \text{Eq. S1}$$

All the samples were analysed in powder form and after gentle grinding with mortar and pestle.

The simulated powder patterns were generated using Mercury[5] and the published crystal structures of cellulose I_{α},[6] I_{β},[7] II,[8] III_{I}[9] and III_{II}.[10]

1.9. Raman spectroscopy

Raman images were obtained using a Raman spectrometer (Renishaw, UK). Raman spectra were acquired using a 785 nm wavelength laser (NIR) and 41 mW laser power at the sample for excitation of Raman scattering. The sample was focused with a 50× objective lens (numerical aperture: 0.7, vertical resolution: 1.6 µm) with a lateral resolution of 684 nm. Due to the narrow spectrum acquisition range, each complete spectrum is a composite of three individual spectra centred at 590, 1090 and 1490 cm^{-1} respectively. Each individual spectrum was obtained from 10 exposures of 20 seconds each. Overlap of the individual spectra enabled their normalisation to produce the complete spectrum. Three complete spectra were acquired for each sample. Raman bands, background fluorescence and photon spikes were fitted for each complete spectrum using Lorentzian curve functions in Fityk,[11] enabling deconvolution of each band and precise identification of their wavelengths and relative intensities. The spectrum for

each sample presented in the paper is the average of the three reconstructed spectra produced from the Lorentzian functions after noise (photon spikes, background fluorescence) removal.

1.10. Characterisation of cellodextrins by solution and solid-state NMR

^{1}H-^{13}C cross-polarisation solid-state NMR experiments of the single 2-, 3- and 6- monofluorinated EpC 10 wt% and multi-6F-EpC 25 wt% hydrogels were performed at 5 °C using a Bruker Avance III spectrometer equipped with a 4 mm triple resonance probe operating at frequencies of 400.2 MHz (^{1}H) and 100.6 MHz (^{13}C). The WPT-CP experiment was carried out using a T_2 filter and mixing time of 2 and 16 ms, respectively. The WPT factors shown in Figure 6 were calculated by normalisation of the peak intensities of the spectrum acquired at 16 ms mixing time against a reference spectrum at 0 ms mixing time. Each gel was packed into a kel-f insert, sealed using a plug and a screw, and spun at 6 kHz. For the powder samples, experiments were carried out at the UK 850 MHz solid state NMR facility (Warwick). All the experiments were run at frequencies of 850.2 MHz (^{1}H), 799.8 MHz (^{19}F) and 213.8 MHz (^{13}C). ^{1}H-decoupled ^{1}H-^{19}F CP (^{1}H-^{19}F{1H}), ^{1}H-^{13}C CP with ^{1}H and ^{19}F decoupling (^{1}H-^{13}C{^{1}H,^{19}F}), and ^{19}F-^{13}C CP with ^{1}H and ^{19}F decoupling (^{19}F-^{13}C{^{1}H,^{19}F}) experiments were run using a 2.5 mm HFX H13894 probe, at 15 kHz MAS spinning and 12 ± 3 °C. A field strength of 67 or 83 kHz was employed for ^{1}H decoupling. The CP contact time was optimised to 1 ms and a relaxation delay of 3 s was employed. A 90° pulse of 4 and 3 μs was used for ^{1}H and ^{19}F, respectively. ^{1}H-undecoupled ^{19}F spectra with ^{19}F background suppression (^{19}F-bs), and ^{1}H-decoupled and ^{19}F-undecoupled ^{1}H-^{13}C CP (^{1}H-^{13}C{1H}) experiments were run using a 1.3 mm X/Y/H-F H13863 probe, at 60 kHz MAS spinning and 27 ± 3 °C. The CP contact time was optimised to 3 ms and a relaxation delay of 3 s was employed. A 90° pulse of 1.5 μs was employed for ^{1}H and ^{19}F. All spectra were referenced with respect to TMS.

The NMR characterisation of EpC dispersed in D_2O was carried out using a Bruker Avance I spectrometer equipped with a 5 mm probe operating at frequencies of 499.7 MHz (^{1}H) and 125.7 MHz (^{13}C). Around 600 μL of dispersion (2 w/V%) in 99.9% D_2O (Sigma-Aldrich®) was pipetted into a 5 mm NMR tube at room temperature. Both phase-sensitive ^{1}H-^{13}C HSQC experiment with ^{1}H-^{13}C correlation via double inept transfer and ^{1}H-^{1}H COSY experiment with multiple quantum filter and gradient ratio for artifact suppression were acquired with 256 increments in the F1 dimension, 8 number of scans and a relaxation delay of 2 s. A ^{13}C DEPT135 experiment with ^{1}H decoupling was acquired using a pulse length of 11.25 μs and 8*k* number of scans.

The solution NMR characterisation of a 0.5 wt% dispersion of multi-6F-EpC (**7**) was carried out using a Bruker Avance III spectrometer equipped with a 5 mm inverse triple-resonance probe operating at frequencies of 800.2 MHz (^{1}H) and 201.2 (^{13}C). Phase-sensitive ^{1}H-^{13}C HSQC experiment with ^{1}H-^{13}C correlation via double inept transfer was acquired at 293K with 128 increments in the F1 dimension, 8 number of scans and a relaxation delay of 3 s. A ^{1}H-^{1}H COSY experiment with multiple quantum filter and gradients was acquired at 5 °C with 256 increments in the F1 dimension, 12 number of scans and a relaxation delay of 2 seconds. Finally, a ^{13}C DEPT135 experiment with ^{1}H decoupling was acquired using a Bruker Avance Neo spectrometer equipped with a cryoprobe operating at frequencies of 600.2 MHz (^{1}H) and 150.9 MHz (^{13}C). A relaxation delay of 1 s was used and 30*k* scans were registered.

2. Results

2.1. Enzymatic synthesis of fluorinated cellobioses and cellodextrins

2.1.1. Nuclear magnetic resonance (NMR) characterisation

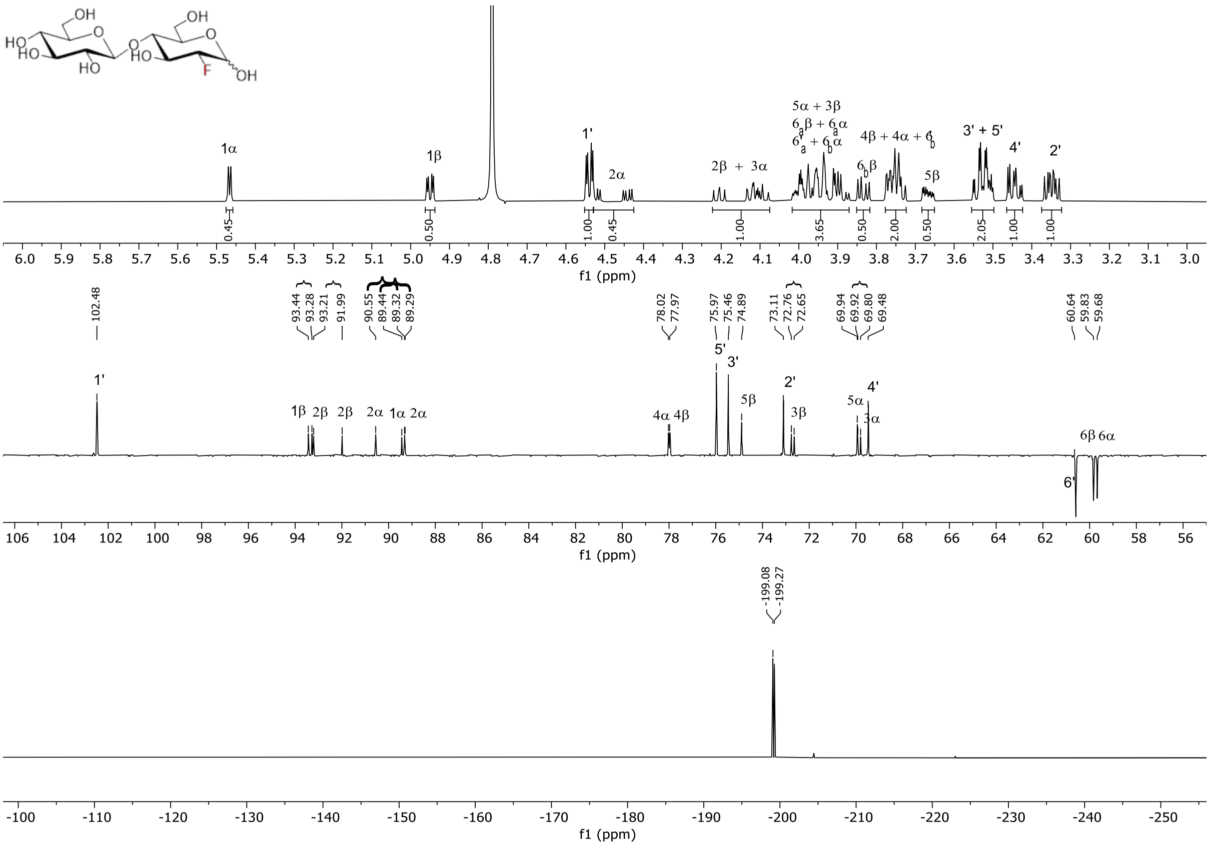

Figure S1. ^{1}H, ^{13}C and $^{19}F\{^{1}H\}$ NMR of 2-deoxy-2-fluoro-cellobiose (**1**).

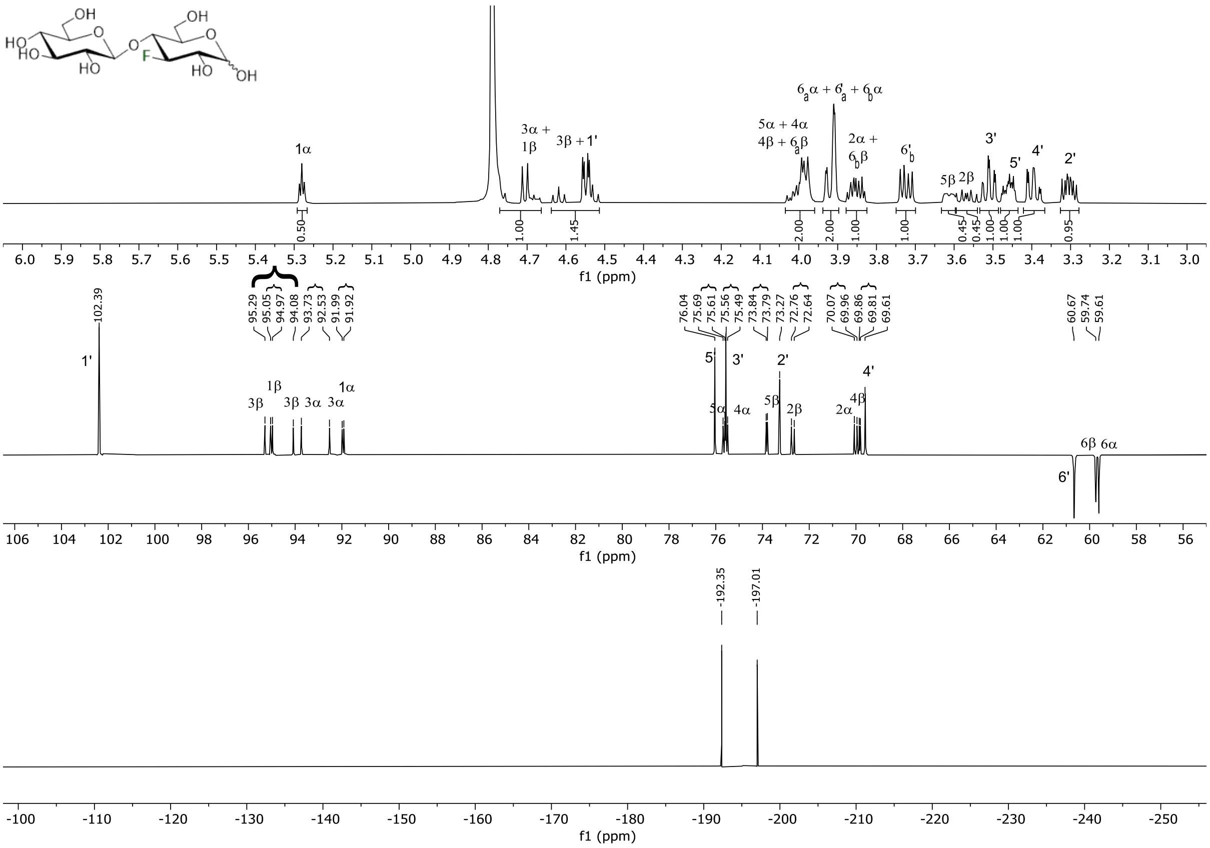

Figure S2. ^{1}H, ^{13}C and $^{19}F\{^{1}H\}$ NMR of 3-deoxy-3-fluoro-cellobiose (**2**).

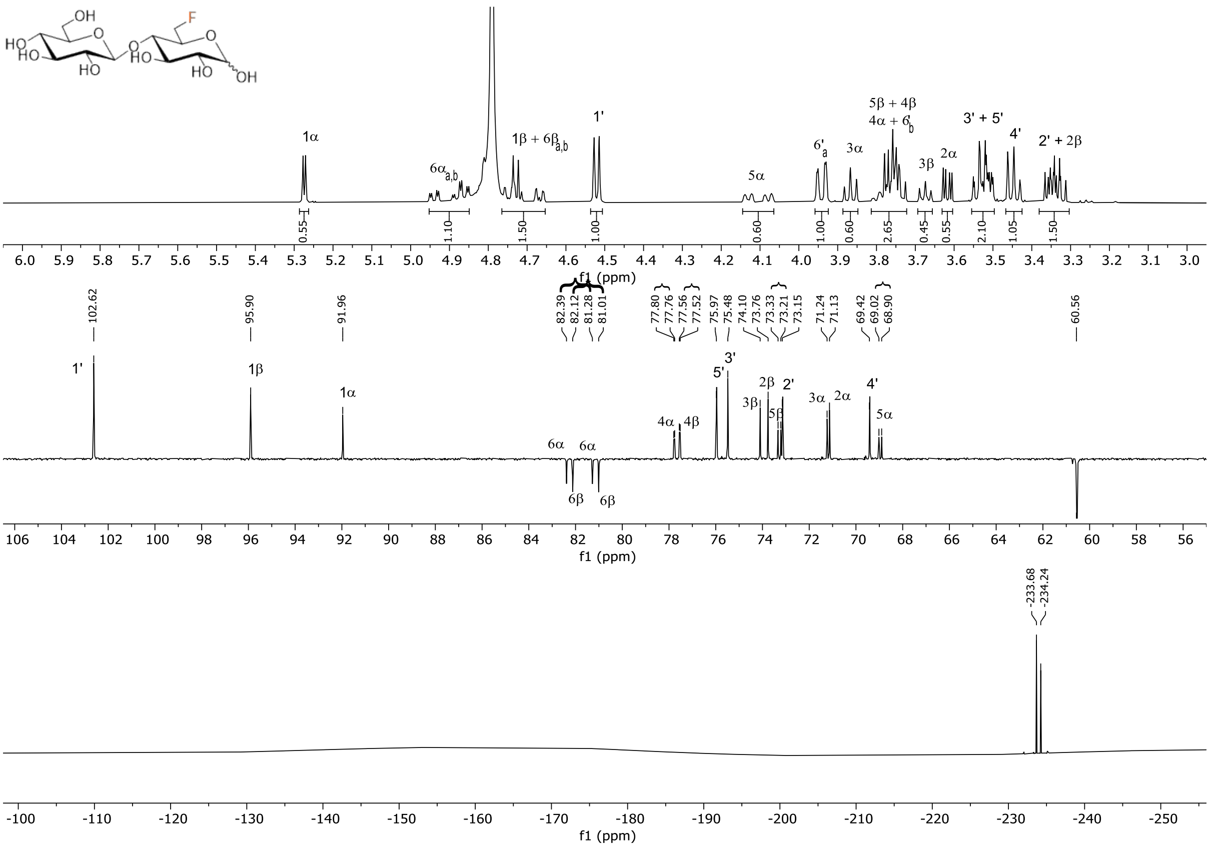

Figure S3. ^{1}H, ^{13}C and $^{19}F\{^{1}H\}$ NMR of 6-deoxy-6-fluoro-cellobiose (**3**).

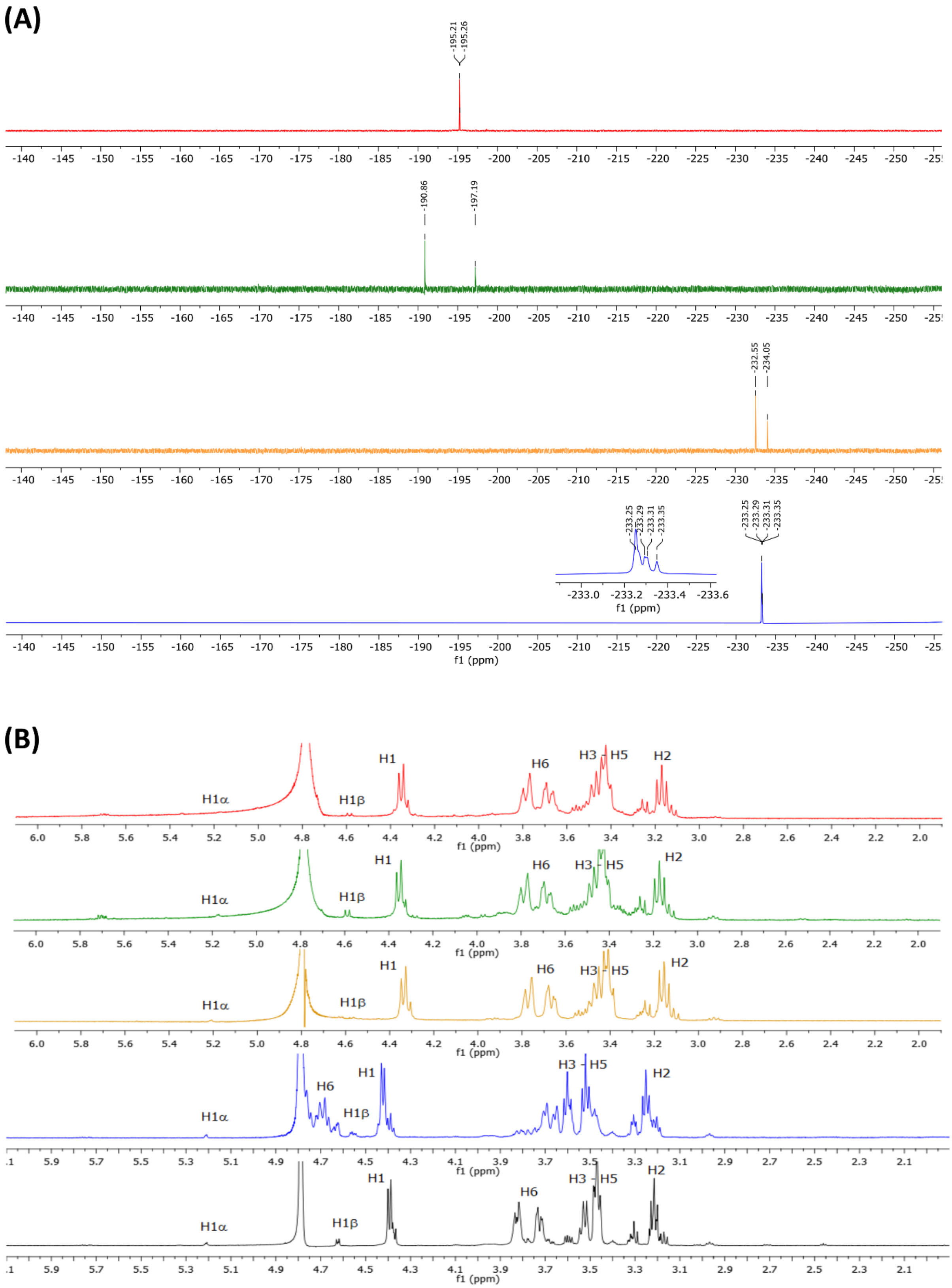

Figure S4. $^{19}F\{^1H\}$NMR (**A**) and ^1H NMR (**B**) spectra in 1 M NaOD of 2-Epc (red), 3F-EpC (green), 6F-EpC (orange), multi-6F-EpC (blue) and EpC (black).

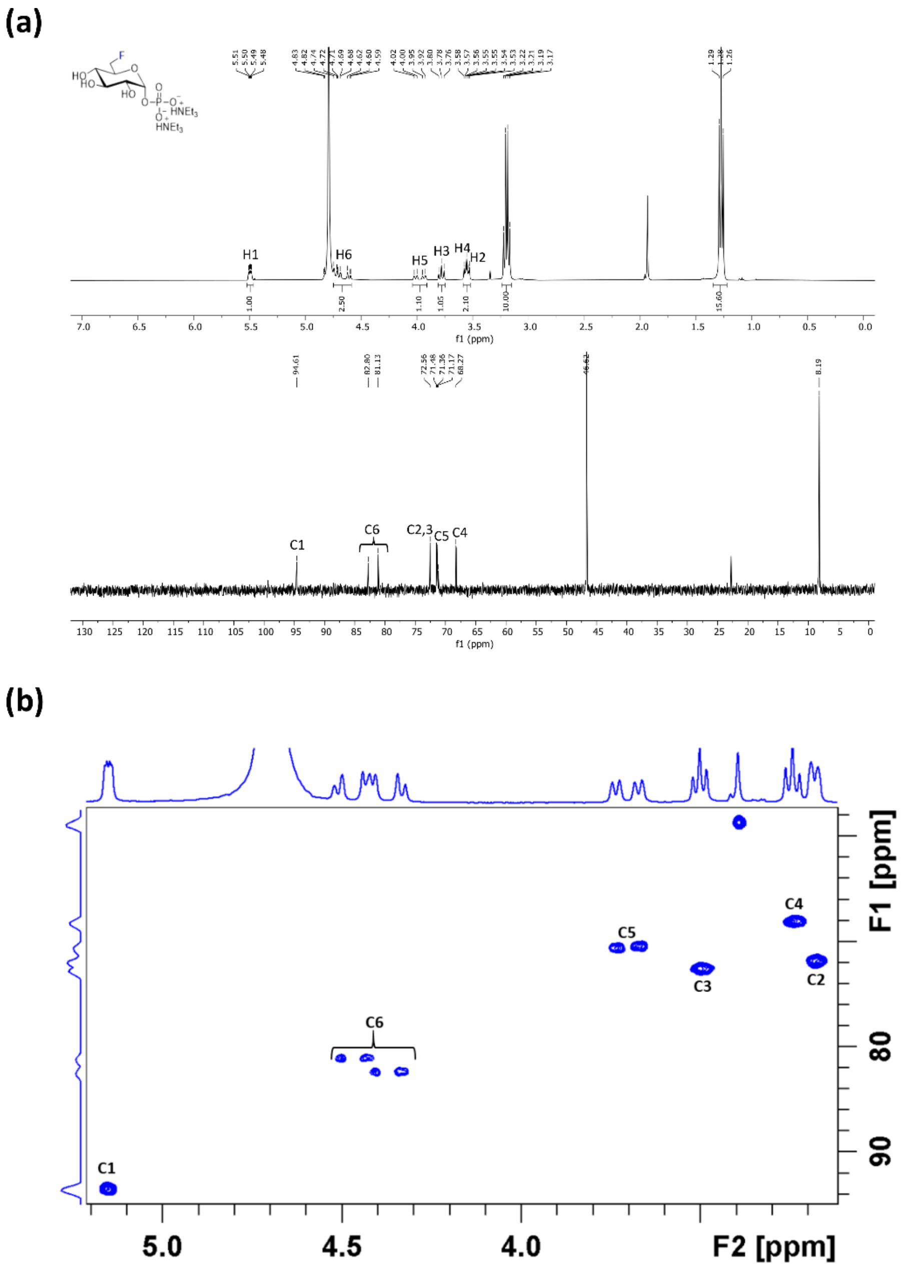

Figure S5. **(a)** ^{1}H (top) and ^{13}C (bottom) in D_2O, and **(b)** ^{1}H-^{13}C HSQC NMR spectra of 6-deoxy-6-fluoro-α-D-glucose 1-phosphate (triethylammonium salt) 2mM in Tris-d_{11} pH 7.4 (25 mM, NaCl 100 mM). Peak assignment is shown.

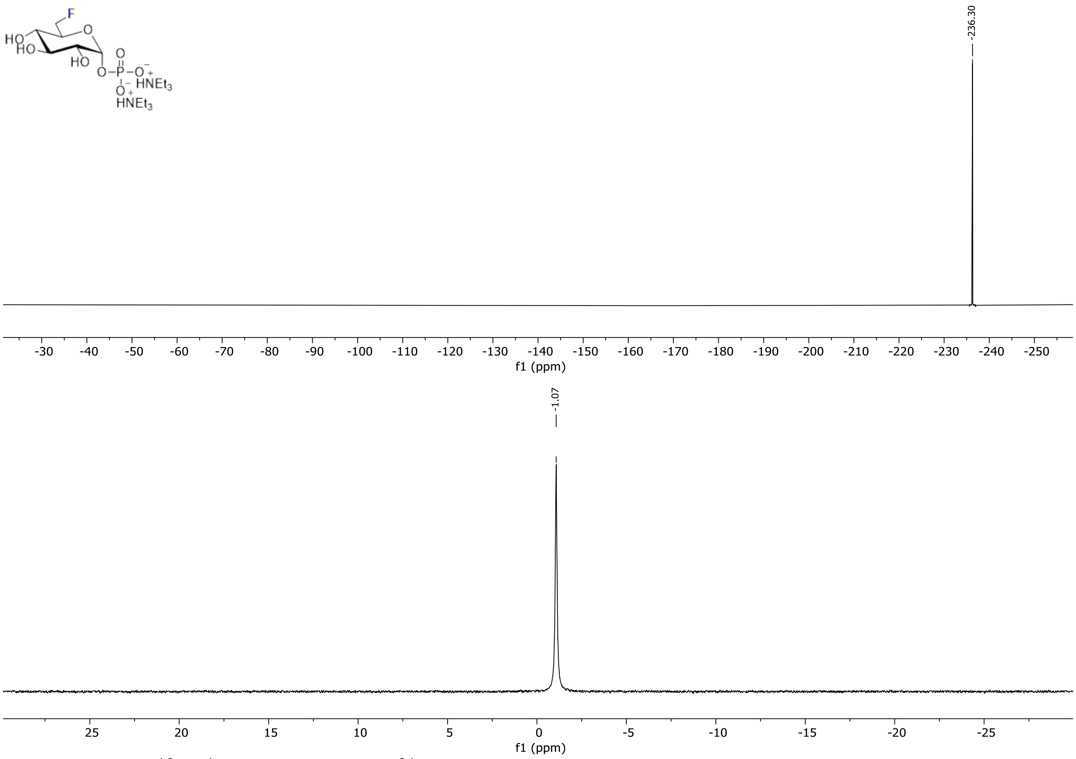

Figure S6. $^{19}F\{^1H\}$ (top) and ^{31}P (bottom) NMR of 6-deoxy-6-fluoro-α-D-glucose 1-phosphate (triethylammonium salt).

2.1.2. Matrix-assisted laser desorption ionisation time-of-flight mass spectrometry (MALDI-TOF MS) characterisation

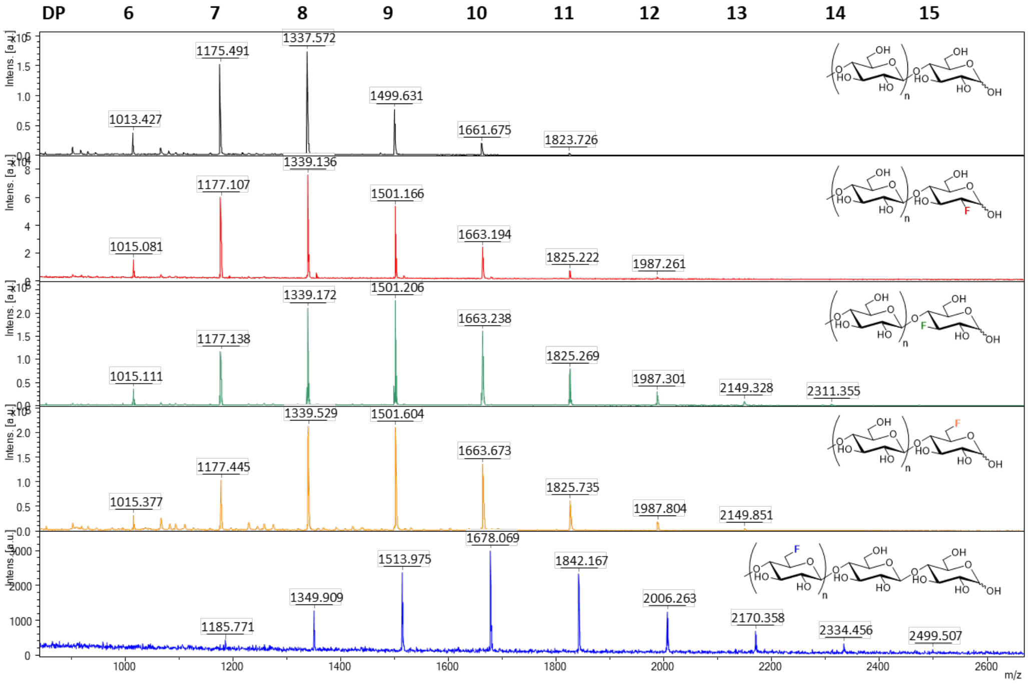

Figure S7. MALDI-TOF spectra comparison between enzymatically produced cellodextrin (EpC, **8**, black line), 2-, 3- and 6-monofluorinated cellodextrins (2F-EpC, **4**, red line; 3F-EpC, **5**, green line; and 6F-EpC, **6**, orange line) and multiply 6-fluorinated cellodextrin (multi-6F-EpC, **7**, blue line).

2.2. Morphological characterisation

2.2.1. Transmission electron microscopy (TEM) characterisation

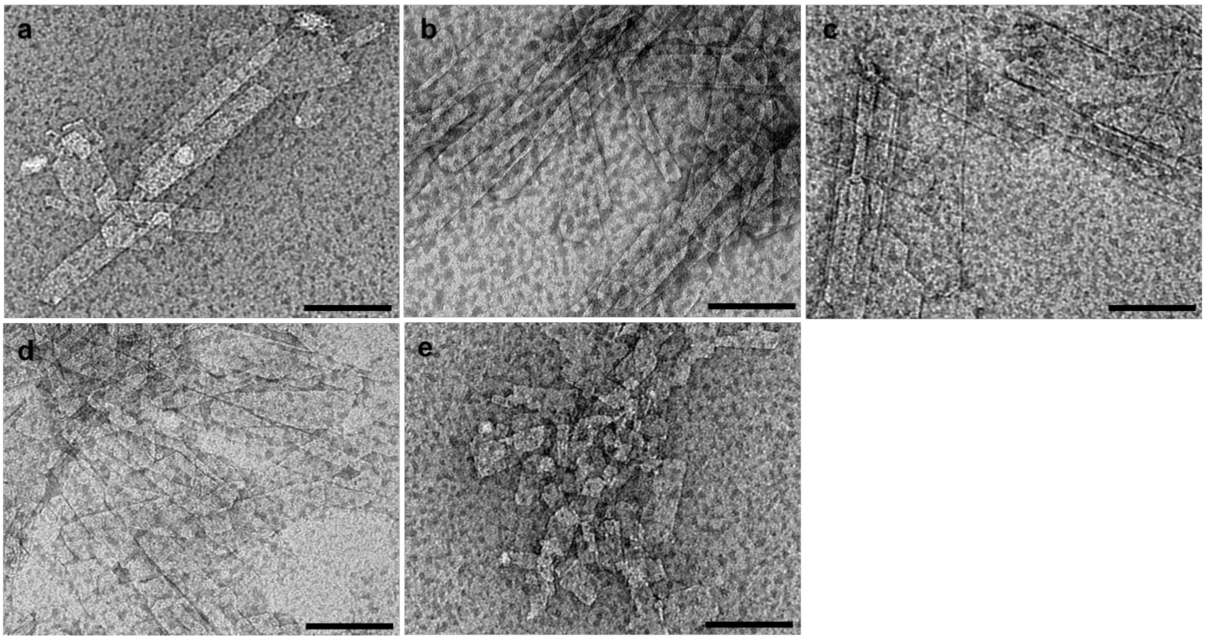

Figure S8. TEM images of enzymatically produced cellodextrin EpC (**a**) and enzymatically produced fluorinated cellodextrins 2F-EpC (**b**), 3F-EpC (**c**), 6F-EpC (**d**) multi-6F-EpC (**e**) negatively stained with 2% uranyl acetate. Scale bars correspond to 100 nm.

2.3. Long-range structural characterisation

2.3.1. Powder X-ray diffraction (PXRD) patterns

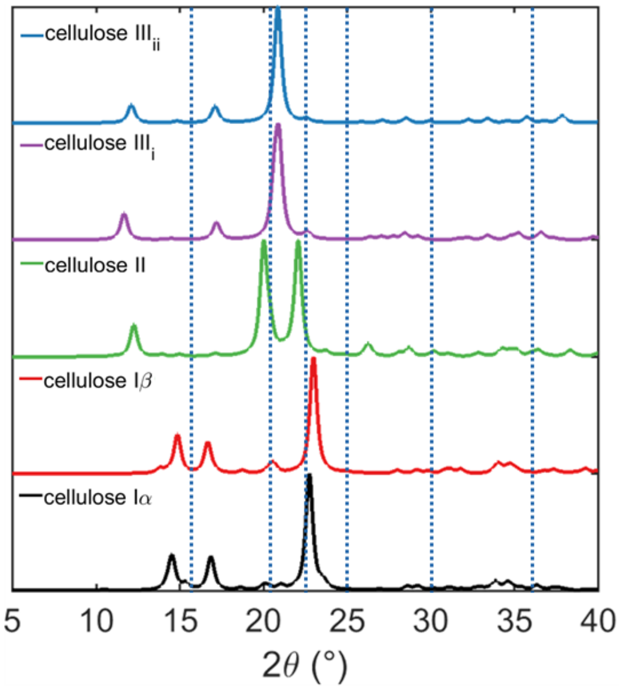

Figure S9. Predicted diffraction patterns for cellulose types I$_\alpha$ (black),[6] I$_\beta$ (red),[7] II (green),[8] III$_I$ (purple)[9] and III$_{II}$ (blue).[10] The patterns were generated using Mercury with FWHM set to 0.5. The blue dashed lines represent the experimental diffraction angles obtained for multi-6F-EpC (the diffraction pattern is reported in the main text, Figure 4b).

Table S1. Comparison of the predicted *2Θ*(°) and *d-spacing* values for cellulose II, III_I and III_{II} with the experimental values obtained for multi-6F-EpC (**7**). Peak indices refer only to cellulose II PXRD pattern (Figure 3).

	**PEAK 1** $(1\bar{1}0)$		**PEAK 2** (110)		**PEAK 3** (020)	
	2Θ(°)	*d-spacing (nm)*	*2Θ*(°)	*d-spacing (nm)*	*2Θ*(°)	*d-spacing (nm)*
**cellulose II**	12.24	0.722	20.02	0.443	22.08	0.402
**cellulose** $\mathbf{III_I}$	11.66	0.758	17.18	0.516	20.88	0.425
**cellulose** $\mathbf{III_{II}}$	12.1	0.731	17.1	0.518	20.86	0.425
**multi-6F-EpC**	15.62	0.567	22.56	0.394		

2.4. Molecular characterisation: local structure

2.4.1. Raman spectroscopy

Table S2. Raman spectroscopy band positions for EpC, 2F-EpC, 3F-EpC, 6F-EpC, and multi-6F-EpC samples.

Band no.	Sample band centre [cm^{-1}]				
	EpC	2F-EpC	3F-EpC	6F-EpC	multi-6F-EpC
1	337	337	338	340	-
2	354	354	354	354	351
3	379	379	379	379	370
4	398	-	-	399	-
5	415	415	416	415	416
6	-	-	-	-	437
7	447	448	448	447	-
8	462	463	463	463	-
9	-	-	-	-	480
10		491	487	493	495
11	519	519	520	519	-
12	576	576	576	576	-
13	637	-	-	-	-
14	-	695	-	-	-
15	844	-	-	844	845
16	867	-	-	867	867
17	896	897	897	896	896
18	-	-	-	-	924
19	969	969	973	969	975
20	1002	-	-	999	1003
21	1029	1030	-	1030	-
22	1064	1065	1064	1065	-
23	1097	1098	1098	1097	1088
24	1123	1122	1124	1122	1127
25	1147	1148	1147	1147	-
26	1235	1235	1232	1234	1239
27	1265	1265	1265	1265	1268
28	1314	1312	1312	1308	1304
29	1336	1337	1336	1336	-
30	-	-	-	1344	1340
31	1368	1361	1369	1362	-
32	-	1374	-	1375	1379
33	-	-	-	1396	-
34	1407	1404	1409	1414	1424
35	1462	1462	1461	1461	1451

2.4.2. Solid-state and solution state NMR spectroscopy

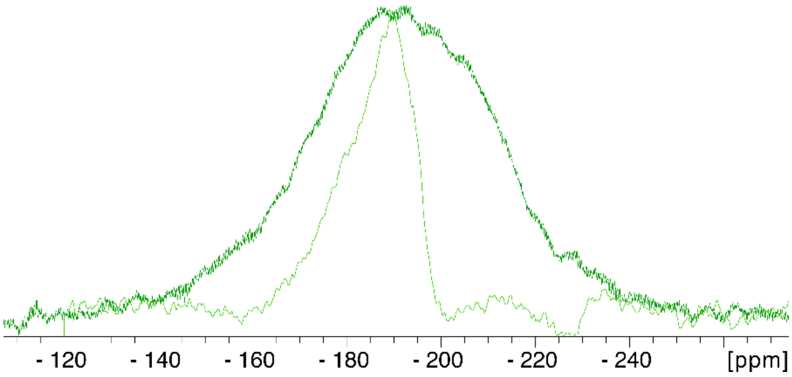

Figure S10. ^{19}F NMR spectrum of 3F-EpC powder acquired with (dark green) and without (light green) ^{1}H decoupling at 15 kHz and 60 kHz MAS rate, respectively, and 800 MHz ^{19}F frequency. Note the more efficient dipolar decoupling at fast MAS.

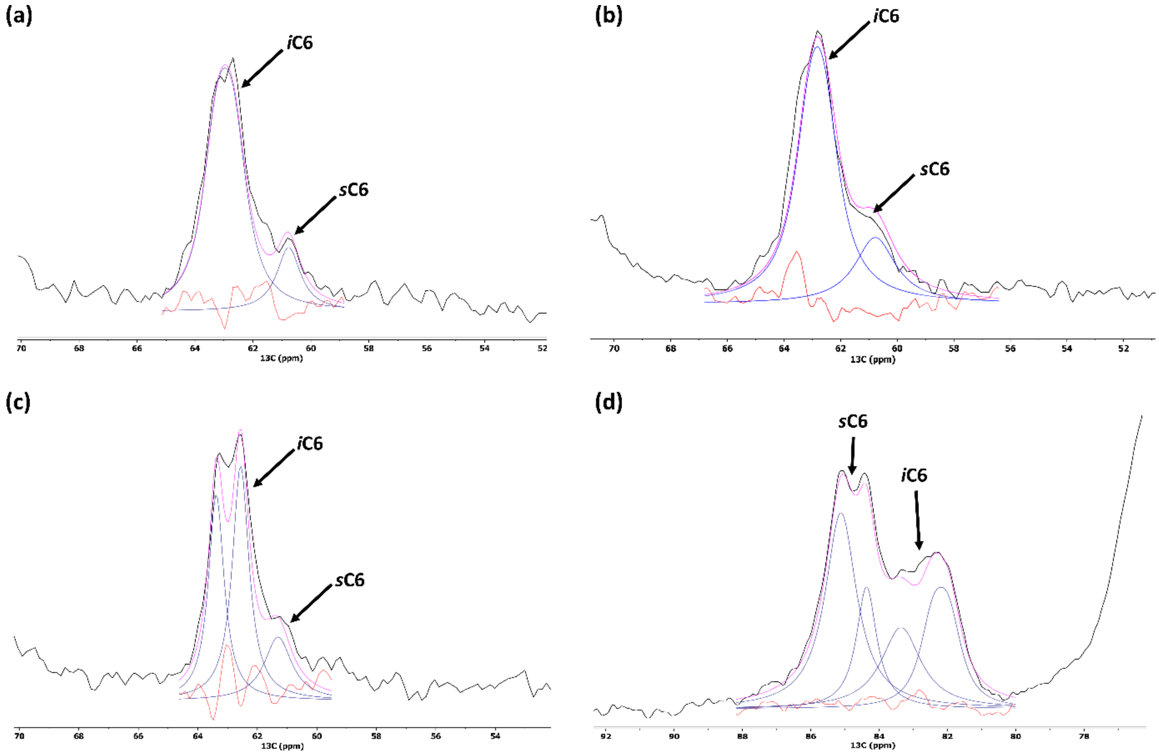

Figure S11. Spectral deconvolution of the *s*C6 and *i*C6 peaks of ^{1}H-^{13}C CP spectra of 2F-EpC (a), 3F-EpC (b), 6F-EpC (c) and multi-6F-EpC (d). The experimental and deconvoluted spectra are shown with black and blue lines, respectively. The sum and residuals spectra are shown in pink and red, respectively.

Table S3. Relative surface area (RSA) values derived from deconvolution of the sC6 and iC6 peaks of ^{1}H-^{13}C CP spectra of the three monofluorinated EpCs and multi-6-EpC powders. RSA were calculated as the ratio of the *s*C6 peak area against the total C6 area (*s*C6 + *i*C6).

	**Relative surface area (%)**
**2F-EpC**	16
**3F-EpC**	23
**6F-EpC**	20
**multi-6F-EpC**	54

The ^{1}H-^{13}C CP/MAS spectrum of multi-6F-EpC (**7**) was compared to all previously reported cellulose allomorphs to identify differences and similarities on ^{13}C chemical shifts (Tables S4, S5).

Table S4. ^{13}C chemical shifts reported in the literature for all the knowns cellulose allomorphs.
* Experimental values obtained in-house for enzymatically produced cellulose (EpC).

**Cellulose allomorph**	**Chemical shift (ppm)**		
	**C1**	**C4**	**C6**
cellulose Iα (Glaucocystis)[12]	105	89.7, 88.8	65.3
cellulose Iβ (tunicin)[12]	105.7, 103.9	88.7, 88.0	65.5, 64.9
cellulose II[10]	107.0, 104.7	88.6, 87.4	62.9, 62.2
*cellulose II (EpC)**	107.0, 104.8	88.6, 87.5	62.9, 62.2
cellulose III_I[12]	104.8	87.8	62.3
cellulose III_{II}[10]	106.5, 105.7	88.5, 87.3	62.5, 62.1

Table S5. ^{13}C chemical shifts of multi-6F-EpC (**7**) acquired in solid-state (top) and solution state (bottom) NMR.

**Chemical shifts (ppm)**					
Solid-state NMR					
**C1**	***s*C6**	***i*C6c**	***s*C2,3,4,5**	***i*C2,3,4,5**	**C6***
104.8	84.2, 83.5	81.9	75.2	73.1	62.6
Solution state NMR					
***s*C1**	***s*C6**	***s*C4***	***s*C2,3,4,5**	***s*C6***	
104.8	84.3, 83.3	79.9	76.0, 75.3	62.1	

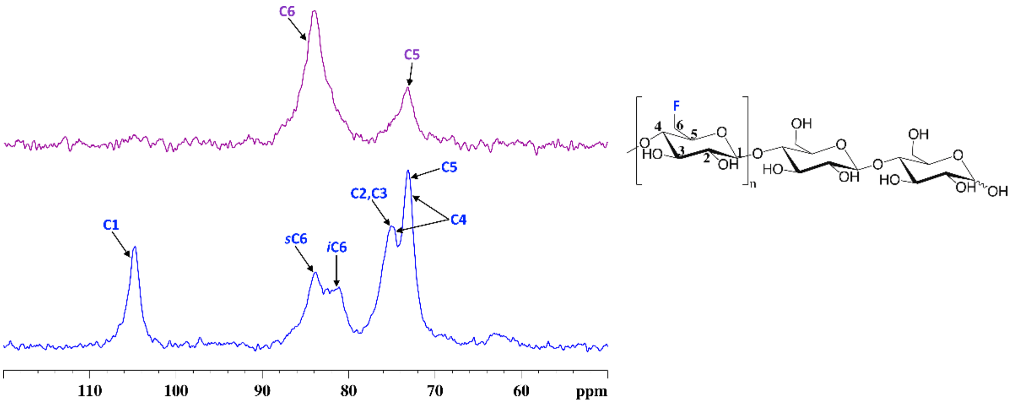

Figure S12. Comparison of the ^{1}H-^{13}C CP (blue) and ^{19}F{^{1}H}-^{13}C CP (purple) NMR spectra of multi-6F-EpC powder acquired at room temperature, 15 kHz MAS rate and a ^{13}C frequency of 212.5 MHz.

The high-resolution solution NMR spectra (COSY and HSQC, Figure S13a) obtained for a diluted dispersion of EpC (**8**) enabled the detailed assignment of the two anomeric spin systems and the internal and non-reducing terminal protons. On the other hand, the higher presence of chemical environments in multi-6F-EpC (**7**) complicated the full spectral assignment (COSY and HSQC, Figure S13b), but we could still assign the β-spin system (belonging to the cellobiose unit) and the 6-fluorinated non-reducing terminal (*nr*).

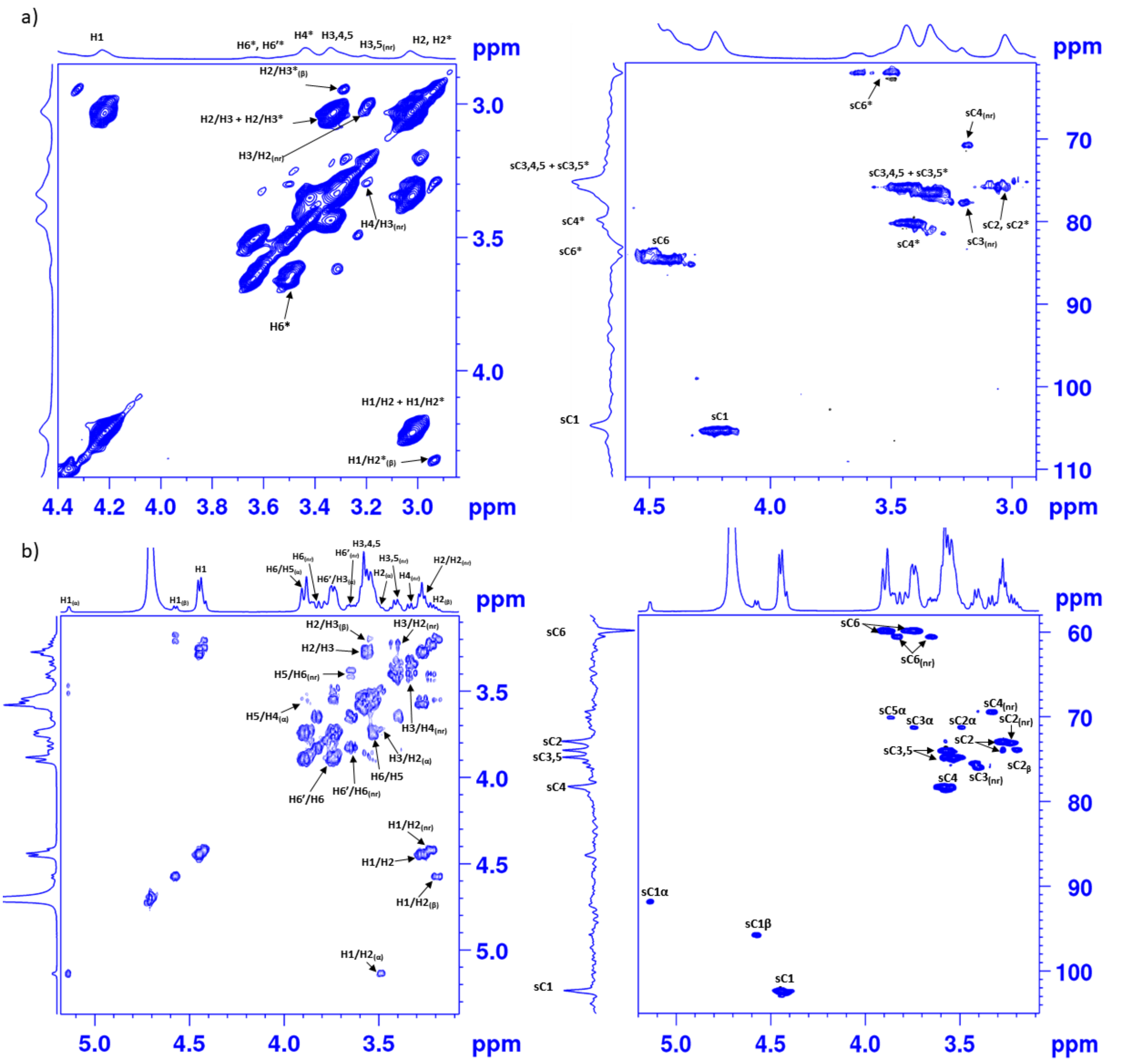

Figure S13. a) ^1H-^1H COSY (left) and ^1H-^{13}C HSQC (right) of a 0.5 wt% dispersion of multi-6F-EpC (**7**). **b)** ^1H-^1H COSY (left) and ^1H-^{13}C HSQC (right) of a 2 wt% dispersion of EpC (**8**). The F1 projection of the ^1H-^{13}C HSQC spectrum in **b** corresponds to a ^{13}C DEPT135 spectrum of the same sample. The cross-peaks corresponding to the reducing end glucose residue are indicated using the α and β labels, whereas the *nr* abbreviation is used for the non-reducing terminal. The peaks corresponding to the cellobiose moiety in multi-6F-EpC (**7**) are indicated with an asterisks (*).

To characterise the internal dynamics of these materials, ^1H-^{13}C CP/MAS NMR experiments at varying contact times were carried out for EpC and multi-6F-EpC (Figure S14). Very similar build-up curves were obtained for all carbon peaks in both powdered samples, which suggest that, at the molecular level, the CP-observable domains (i.e. rigid) of EpC and multi-6F-EpC nanofibrils present very similar dynamics.

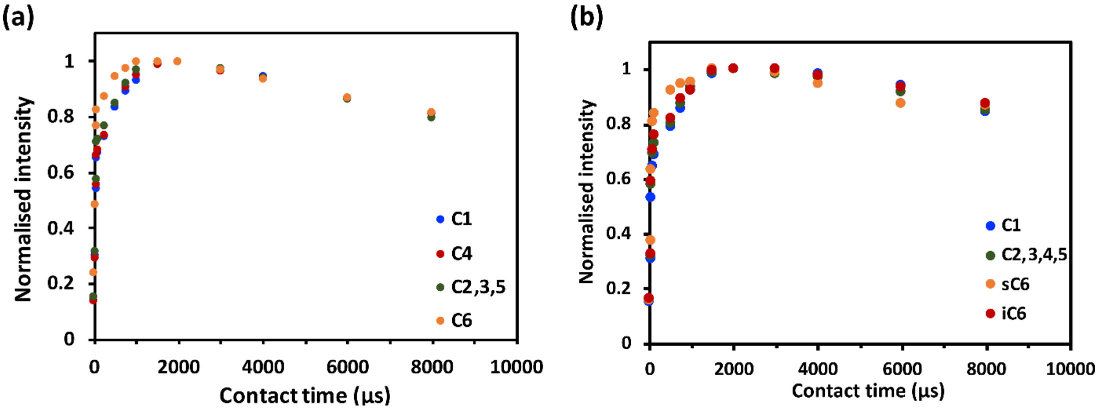

Figure S14. ^{1}H-^{13}C CP build-up curves of EpC (a) and multi-6F-EpC (b) powdered samples acquired at 25 °C, 12 kHz MAS rate and a ^{13}C frequency of 100.6 MHz.

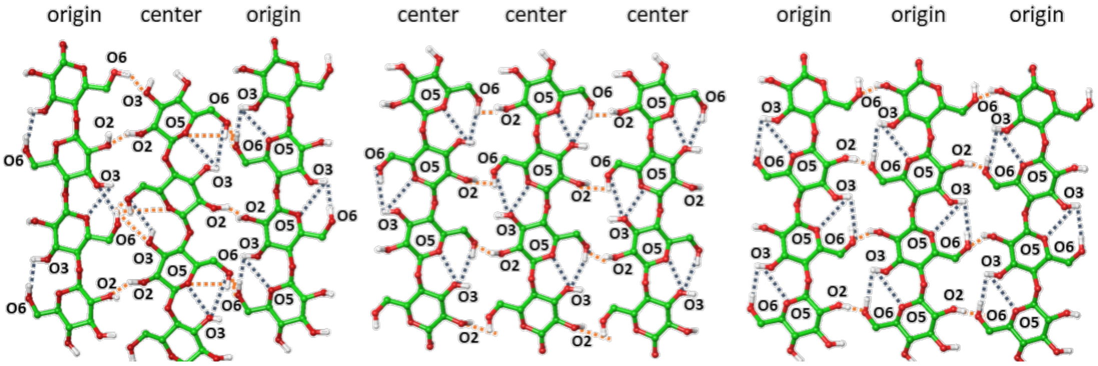

Figure S15. 3D model of the crystalline packing of cellulose II allomorph based on the origin-center-origin (o-c-o), center-center-center (c-c-c) and origin-origin-origin (o-o-o) chains, as described by Langan *et al.*[14] The intra-chain and inter-sheet hydrogen bonds are shown as grey and orange dashes, respectively.

References

1 E. C. O'Neill, G. Pergolizzi, C. E. M. Stevenson, D. M. Lawson, S. A. Nepogodiev and R. A. Field, *Carbohydr. Res.*, 2017, **451**, 118–132.
2 M. A. Arai, Y. Yamaguchi and M. Ishibashi, *Org. Biomol. Chem.*, 2017, **15**, 5025–5032.
3 S. G. Withers, M. D. Percival and I. P. Street, *Carbohydr. Res.*, 1989, **187**, 43–66.
4 J. S. Zhu, N. E. McCormick, S. C. Timmons and D. L. Jakeman, *J. Org. Chem.*, 2016, **81**, 8816–8825.
5 C. F. Macrae, I. J. Bruno, J. A. Chisholm, P. R. Edgington, P. McCabe, E. Pidcock, L. Rodriguez-Monge, R. Taylor, J. Van De Streek and P. A. Wood, *J. Appl. Crystallogr.*, 2008, **41**, 466–470.
6 Y. Nishiyama, J. Sugiyama, H. Chanzy and P. Langan, *J. Am. Chem. Soc.*, 2003, **125**, 14300–14306.
7 Y. Nishiyama, P. Langan and H. Chanzy, *J. Am. Chem. Soc.*, 2002, **124**, 9074–9082.
8 P. Langan, Y. Nishiyama and H. Chanzy, *Biomacromolecules*, 2001, **2**, 410–416.
9 M. Wada, H. Chanzy, Y. Nishiyama and P. Langan, *Macromolecules*, 2004, **37**, 8548–8555.
10 M. Wada, L. Heux, Y. Nishiyama and P. Langan, *Biomacromolecules*, 2009, **10**, 302–309.
11 M. Wojdyr, *J. Appl. Crystallogr.*, 2010, **43**, 1126–1128.
12 M. Wada, L. Heux, A. Isogai, Y. Nishiyama, H. Chanzy and J. Sugiyama, *Macromolecules*, 2001, **34**, 1237–1243.
13 W. Kolodziejski and J. Klinowski, *Chem. Rev.*, 2002, **102**, 613–628.
14 P. Langan, Y. Nishiyama and H. Chanzy, *J. Am. Chem. Soc.*, 1999, **121**, 9940–9946.
